# Serum Metabolomics for Prognostic Stratification in Resected Advanced-Stage Oral Cavity Cancer

**DOI:** 10.1001/jamaoto.2025.4267

**Published:** 2025-12-04

**Authors:** Eric Yi-Liang Shen, Li-Yu Lee, Shu-Hang Ng, Chien-Yu Lin, Hung-Ming Wang, Chia-Hsun Hsieh, Chih-Hua Yeh, Shiang-Fu Huang, Chung-Jan Kang, Tzu-Chen Yen, Nai-Ming Cheng, Chun-Ta Liao

**Affiliations:** 1 Department of Radiation Oncology and Proton Therapy Center, Chang Gung Memorial Hospital, Linkou and Chang Gung University, Taoyuan City, Taiwan, Republic of China; 2 Clinical Metabolomics Core Laboratory, Chang Gung Memorial Hospital, Linkou, Taoyuan City, Taiwan, Republic of China; 3 Department of Pathology, Chang Gung Memorial Hospital and Chang Gung University, Taoyuan, Taiwan, Republic of China; 4 Department of Diagnostic Radiology, Chang Gung Memorial Hospital and Chang Gung University, Taoyuan, Taiwan, Republic of China; 5 Department of Radiation Oncology, Chang Gung Memorial Hospital and Chang Gung University, Taoyuan, Taiwan, Republic of China; 6 Department of Medical Oncology, Chang Gung Memorial Hospital and Chang Gung University, Taoyuan, Taiwan, Republic of China; 7 Department of Otorhinolaryngology, Head and Neck Surgery, Chang Gung Memorial Hospital and Chang Gung University, Taoyuan, Taiwan, Republic of China; 8 Department of Nuclear Medicine and Molecular Imaging Center, Chang Gung Memorial Hospital and Chang Gung University, Taoyuan, Taiwan, Republic of China

## Abstract

**Importance:**

Improved methods are needed to predict recurrence in oral cavity squamous cell carcinoma (OCSCC). However, to date, no metabolome studies have fully explored the prediction of OCSCC relapse patterns and survival.

**Objective:**

To identify serum metabolites associated with OCSCC recurrence and develop and validate a prognostic scoring system.

**Design, Setting, and Participants:**

This retrospective cohort study was conducted at a single tertiary academic center and enrolled patients with histologically confirmed, surgically resected first primary advanced-stage OCSCC from betel quid–chewing areas. Patients underwent primary surgery between February 2007 and May 2018, with follow-up data systematically collected through a prospectively maintained institutional registry. Data were analyzed from December 2024 to September 2025.

**Exposures:**

Preoperative serum metabolomic profiling was performed using liquid chromatography–mass spectrometry.

**Main Outcomes and Measures:**

Identification of metabolites predictive of relapse and survivals; development and validation of the MetaboScore.

**Results:**

Of 228 included patients, 216 (94.7%) were male, and the mean (SD) age at OCSCC onset was 51.9 (10.8) years. A total of 19 metabolites associated with relapse were included in the MetaboScore. A threshold score of 11 or greater was associated with a higher risk of relapse, including local recurrence (22 of 79 [27.8%] vs 5 of 149 [3.4%]), regional recurrence (19 [24.1%] vs 10 [6.7%]), and distant metastasis (29 [36.7%] vs 21 [14.1%]) compared with scores less than 11. Patients with scores of 11 or greater had worse 5-year disease-free survival (38% [95% CI, 28-51] vs 83% [95% CI, 77-89]; difference, 45 percentage points; 95% CI, 32-57) and disease-specific survival (58% [95% CI, 48-70] vs 85% [95% CI, 79-91]; difference, 27 percentage points; 95% CI, 14-40). Multivariable analysis confirmed the independent prognostic value of the MetaboScore (local recurrence: hazard ratio [HR], 14.84; 95% CI, 5.45-40.40; regional recurrence: HR, 4.22; 95% CI, 1.94-9.15; distant metastases: HR, 2.53; 95% CI, 1.42-4.50; disease-free survival: HR, 4.40; 95% CI, 2.71-7.15; disease-specific survival: HR, 3.04; 95% CI, 1.76-5.25). Notably, the MetaboScore provided additional prognostic information beyond pN3b status.

**Conclusions and Relevance:**

In this cohort study, a high MetaboScore was associated with local recurrence, regional recurrence, and distant metastasis, even after conventional staging was applied, in OCSCC. Its derivation from a cohort predominantly composed of male patients with betel quid exposure and advanced-stage disease warrants caution. Prospective validation in more diverse populations comprising different etiologies, disease stages, and demographic characteristics will be essential before broad clinical implementation.

## Key Points

**Question:**

Can serum metabolomics identify biomarkers predictive of relapse and survival in resected oral cavity squamous cell carcinoma (OCSCC)?

**Findings:**

In this cohort study including 228 patients with advanced-stage disease and a high prevalence of betel quid exposure, a novel serum metabolite-based prognostic scoring system was developed and validated. Patients with a high score (≥11) demonstrated worse 5-year local control, neck control, distant metastases, disease-free survival, and disease-specific survival compared with those with lower scores (<11).

**Meaning:**

Serum metabolomic profiling enhances prognostic stratification in OCSCC and could inform personalized therapeutic decisions.

## Teaser

This cohort study identifies serum metabolites associated with oral cavity squamous cell carcinoma recurrence and develops and validates a prognostic scoring system.

## Introduction

In recent years, there has been a notable increase in the incidence of oral cavity cancer, contributing significantly to the overall disease burden worldwide.^1^ In 2022, there were more than 389 000 new cases reported globally, resulting in approximately 188 000 deaths.^2^ Among oral malignant tumors, oral cavity squamous cell carcinoma (OCSCC) represents the predominant histological type.^3,4^ Notably, treatment strategies have evolved over time to incorporate a multidisciplinary approach, where surgical resection serves as the cornerstone of therapy, potentially complemented by adjuvant radiotherapy or chemoradiotherapy in the presence of adverse pathological risk factors.^5,6^ Unfortunately, the treatment outcomes for advanced-stage OCSCC remain dismal. The management of OCSCC is complicated by a high rate of postsurgical recurrences, with estimated relapse risks of 30% at 5 years and 37% at 10 years.^7,8^ Despite established risk stratification based on clinicopathological parameters,^6^ current prognostic models inadequately predict individual tumor behavior. This biological heterogeneity underscores the need for more refined risk stratification approaches to optimize therapeutic decision-making and personalized treatment strategies.

Recent advances in metabolomics have enabled researchers to identify novel prognostic biomarkers in patients with malignant neoplasms.^9,10^ Interestingly, previous studies have also highlighted the significant potential of metabolomics in improving the assessment of surgical margins in OCSCC.^11,12^ This innovative approach offers a valuable alternative to traditional intraoperative pathological diagnosis, aiding in the precise determination of clear surgical margins. However, prior metabolomic studies of OCSCC that analyzed saliva or focused primarily on overall survival outcomes,^13,14,15^ and the potential utility of serum metabolite alterations in predicting disease recurrence and enabling prognostic stratification in patients with OCSCC remains largely unexplored. To address this knowledge gap, we designed the current retrospective study of prospectively collected data in a large cohort of resected advanced-stage OCSCC to identify differentially expressed metabolites in the sera of patients with OCSCC with and without disease recurrence, leading to the development of the MetaboScore, a novel numerical prognostic system. We also investigated how integrating the MetaboScore with traditional risk factors could enhance risk stratification.

## Methods

The study was conducted in accordance with the ethical principles outlined in the Declaration of Helsinki. Approval was obtained from the Institutional Review Board of the Chang Gung Medical Foundation, and all participants provided written informed consent prior to the day of surgery. This study followed the Reporting Recommendations for Tumor Marker Prognostic Studies (REMARK) reporting guideline.

### Study Patients

All patients were evaluated at Chang Gung Memorial Hospital, Linkou, Taiwan, from February 2007 to May 2018, with oversight from a multidisciplinary team comprising specialists in surgical oncology, radiation oncology, and medical oncology. Treatment-naive patients with histologically confirmed first primary OCSCC who underwent primary surgical intervention were deemed eligible. The preoperative staging protocol consisted of a comprehensive clinical assessment, radiological evaluation (chest radiography and head and neck computed tomography/magnetic resonance imaging), and ^18^F-fludeoxyglucose–positron emission tomography imaging. All relevant clinicopathological factors, including depth of invasion and extranodal extension, were systematically recorded in an established institutional registry, facilitating accurate revisions from the *American Joint Committee on Cancer* (*AJCC*) *Staging Manual*, *seventh edition* to the *AJCC Staging Manual, eighth edition*. Clinicopathological risk factors were prospectively collected by investigators blinded to the clinical outcomes of the study end points. Two experienced head and neck pathologists (including L.Y.L.) independently reviewed all histopathological data using a dedicated checklist according to the College of American Pathologists Cancer Reporting Protocols recommendation. The primary variables of interest encompassed patient demographic characteristics (age and sex), oral behavioral factors (betel quid chewing and cigarette smoking), pathological staging parameters (pT, pN, and p stage), histopathological risk factors (tumor differentiation, extranodal extension, tumor depth, margin status, bone marrow invasion, skin invasion, perineural invasion, lymphatic invasion, and vascular invasion), as well as treatment modalities. Inconclusive or less relapse-specific pathological features, such as the worst pattern of invasion, which has been predominantly correlated with overall survival,^16^ were not included. Detailed treatment protocols, follow-up surveillance, and salvage therapy approaches are described in the eMethods in Supplement 1.

### Study End Points

The outcomes of interest included the 5-year rates of disease-free survival (DFS), disease-specific survival (DSS), local control, neck control, and distant metastasis, which were calculated as the time from the date of surgery to the date of the event of interest. Censoring occurred at the date of the last follow-up in November 2024.

### Metabolite Biomarker Discovery

Given that fasting duration may significantly influence metabolomic profiles,^17,18^ all venous blood specimens were collected following a minimum 6-hour fasting period on the day of surgery. The details of serum sample processing and mass spectrometry are provided in the eMethods in Supplement 1. Principal component analysis was conducted for each metabolite to identify metabolomic signatures associated with OCSCC relapse. Partial least-squares discriminant analysis was used to enhance the differentiation between patients with relapsing disease and those without. The initial selection of candidate metabolites associated with relapsing disease was performed using univariable analysis and the fold-change (FC) method. Differences between groups were examined using the unpaired *t* test, with metabolites showing a *P* value less than .05 and an FC exceeding 1.1 or below 0.909 considered differentially expressed. The selection of metabolites was further refined using the eXtreme Gradient Boosting (XGBoost) machine learning algorithm. Additional details regarding metabolite annotation methods are available in the eMethods in Supplement 1.

### MetaboScore Development

In line with prior research,^19,20^ optimal cutoff values for metabolites associated with disease relapse were determined using penalized spline regression within a Cox proportional hazards model. The cutoff was identified as the point where the estimated spline function approached zero on the log hazard ratio scale, corresponding to a hazard ratio (HR) of 1. Term plots of the fitted spline functions illustrated the association between each metabolite and HR. Since metabolites were initially selected based on relapse events using *t* tests, FC analysis, and the XGBoost machine learning algorithm, their associations with DFS were validated through Kaplan-Meier survival curves and log-rank tests. Metabolites showing significant associations with DFS were incorporated into the MetaboScore scoring system. For each metabolite predictive of adverse DFS outcomes, a score of 1 was assigned if present in a patient’s serum and 0 if absent. The final MetaboScore was calculated as the unweighted sum of scores for all included metabolites.

### Statistical Analysis

The optimal cutoff value for the MetaboScore was established by analyzing trends in Kaplan-Meier survival curves and results from the log-rank test. To assess the prognostic significance of the MetaboScore alongside traditional risk factors, both univariable and multivariable analyses were conducted, focusing on 5-year local control, neck control, distant metastases, DFS, and DSS rates. Univariable comparisons between groups were performed using the Wald χ^2^ test.^21,22^ Multivariable HRs for the end points of interest were estimated through Cox proportional hazards modeling with a stepwise selection approach.^23,24^ All factors identified in univariable analysis were entered into the multivariable model, and selection was performed using the Akaike information criterion. Statistical analyses were conducted using R version 4.0.3 (The R Foundation) and SPSS version 22 (IBM). Data were analyzed from December 2024 to September 2025. A 2-tailed *P *value less than .05 was considered statistically significant. Additionally, to address concerns regarding potential overfitting or unstable results from stepwise selection, we performed a least absolute shrinkage and selection operator (LASSO)–penalized Cox proportional hazards regression as an alternative variable selection method.^25,26,27^ All candidate covariates were entered into the LASSO model, and 10-fold cross-validation was used to determine the optimal penalty parameter (λ) that minimizes cross-validated partial-likelihood deviance. The λ that gave the minimum deviance and the λ at 1 SE above the minimum were reported, along with the subset of variables whose coefficients remained nonzero under each penalty.

## Results

### Patient Characteristics

The final cohort included 228 patients, with a mean (SD) follow-up period of 86 (55) months from surgery. A total of 216 (94.7%) were male, and the mean (SD) age at OCSCC onset was 51.9 (10.8) years. Most patients were younger than 65 years (200 [87.7%]) and reported high-risk oral behaviors, including betel quid chewing (201 [88.2%]) and cigarette smoking (199 [87.3%]). Most tumors (205 [89.9%]) were advanced stage (pT3 to pT4), with significant nodal involvement (pN3b: 72 [31.6%]). The 5-year rates were 87.4% (95% CI, 82.5-92.5) for local control, 87.3% (95% CI, 82.9-91.9) for neck control, 22.6% (95% CI, 16.9-27.9) for distant metastases, 67.5% (95% CI, 61.5-74.0) for DFS, and 75.4% (95% CI, 69.8-81.3) for DSS. Detailed demographic, clinical characteristics, and relapse patterns are summarized in Table 1 and in the eMethods and eFigure 1 in Supplement 1.

**Table 1.** General Characteristics of Patients With Resected Oral Cavity Squamous Cell Carcinoma, Including Subgroups Stratified by MetaboScore Values

Characteristic	Patients, No. (%)			Difference, percentage points (95% CI)
	Entire cohort (N = 228)	MetaboScore		
		<11 (n = 149)	≥11 (n = 79)	
Sex				
Female	12 (5.3)	8 (5.4)	4 (5.1)	0.3 (−5.7 to 6.3)
Male	216 (94.7)	141 (94.6)	75 (94.9)	
Age at onset, y				
<65	200 (87.7)	127 (85.2)	73 (92.4)	7.2 (−1.0 to 15.3)
≥65	28 (12.3)	22 (14.8)	6 (7.6)	
Betel quid chewing				
No	27 (11.8)	21 (14.1)	6 (7.6)	−6.5 (−14.6 to 1.6)
Yes	201 (88.2)	128 (85.9)	73 (92.4)	
Cigarette smoking				
No	29 (12.7)	20 (13.4)	9 (11.4)	−2.0 (−10.9 to 6.9)
Yes	199 (87.3)	129 (86.6)	70 (88.6)	
Pathological T status				
pT2	23 (10.1)	20 (13.4)	3 (3.8)	−9.6 (−16.5 to −2.7)
pT3-pT4	205 (89.9)	129 (86.6)	76 (96.2)	
Pathological N status				
pN0-pN2	156 (68.4)	108 (72.5)	48 (60.8)	−11.7 (−24.7 to 1.2)
pN3^a^	72 (31.6)	41 (27.5)	31 (39.2)	
Pathological stage				
III	43 (18.9)	34 (22.8)	9 (11.4)	−11.4 (−21.1 to −1.7)
IV	185 (81.1)	115 (77.2)	70 (88.6)	
Tumor differentiation				
Well to moderate	192 (84.2)	125 (83.9)	67 (84.8)	1.4 (−8.2 to 11.1)
Poor	36 (15.8)	24 (16.1)	12 (15.2)	
Tumor depth, mm				
<10	32 (14.0)	26 (17.4)	6 (7.6)	−9.8 (−18.3 to −1.4)
≥10	196 (86.0)	123 (82.6)	73 (92.4)	
Margin status, mm				
≤4	65 (28.5)	33 (22.1)	32 (40.5)	18.4 (5.6 to 31.1)
>4	163 (71.5)	116 (77.9)	47 (59.5)	
Bone marrow invasion				
No	190 (83.3)	122 (81.9)	68 (86.1)	4.2 (−5.6 to 14.0)
Yes	38 (16.7)	27 (18.1)	11 (13.9)	
Skin invasion				
No	202 (88.6)	130 (87.2)	72 (91.1)	3.9 (−4.4 to 12.1)
Yes	26 (11.4)	19 (12.8)	7 (8.9)	
Perineural invasion				
No	105 (46.1)	66 (44.3)	39 (49.4)	5.1 (−8.5 to 18.7)
Yes	123 (53.9)	83 (55.7)	40 (50.6)	
Lymphatic invasion				
No	215 (94.3)	140 (94.0)	75 (94.9)	1.0 (−5.2 to 7.1)
Yes	13 (5.7)	9 (6.0)	4 (5.1)	
Vascular invasion				
No	213 (93.4)	139 (93.3)	74 (93.7)	0.4 (−6.3 to 7.1)
Yes	15 (6.6)	10 (6.7)	5 (6.3)	
Treatment modality				
Surgery	29 (12.7)	21 (14.1)	8 (10.1)	−4.0 (−12.7 to 4.7)
Surgery plus RT/CRT	199 (87.3)	128 (85.9)	71 (89.9)	

Abbreviations: CRT, chemoradiotherapy; RT, radiotherapy.

^a^ All patients with pN3 disease had extranodal extension (pN3b); no patients had pN3a disease (single metastatic lymph node larger than 6 cm without extranodal extension).

### Identification of Metabolites Associated With OCSCC Relapse

**Figure 1.**
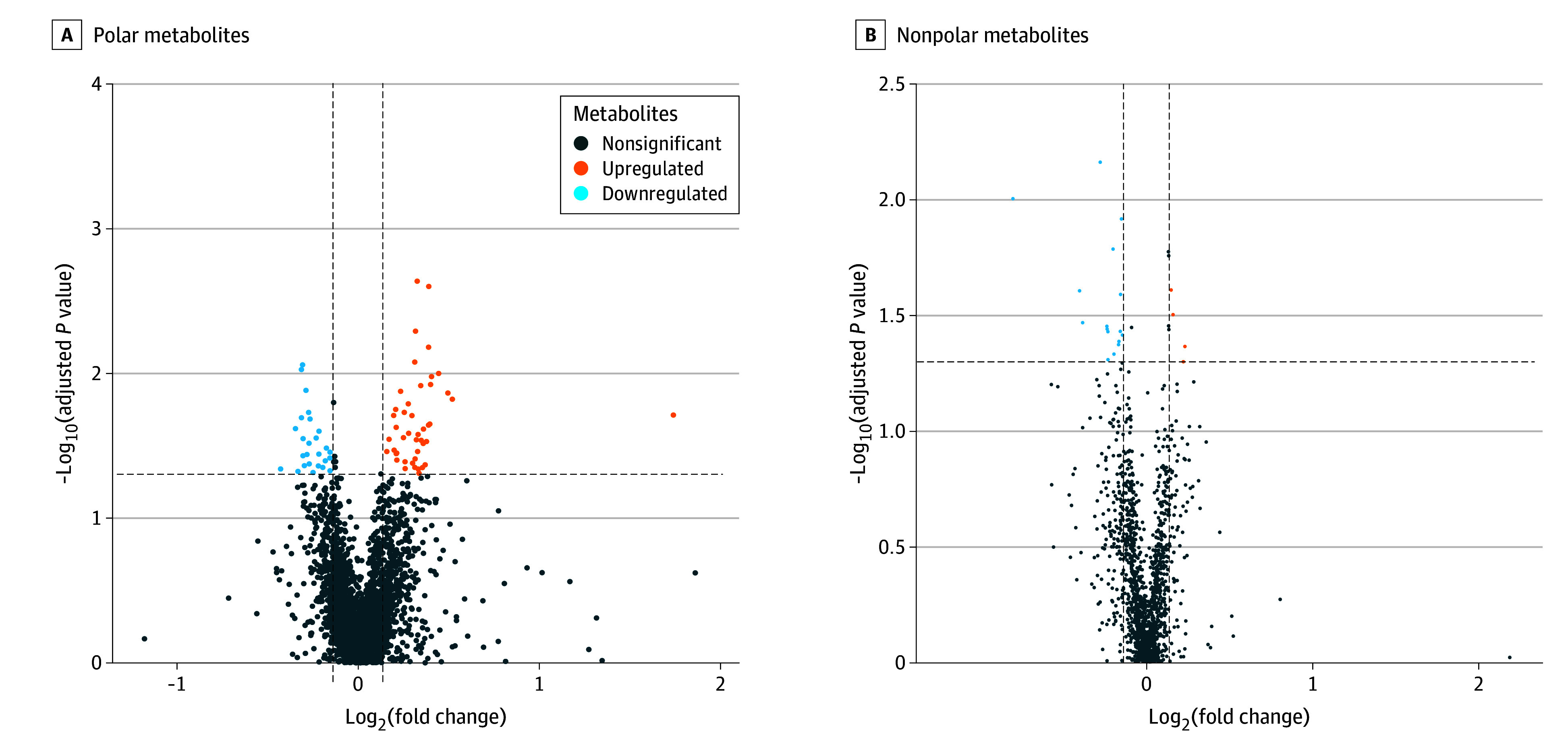
Volcano Plots of Polar and Nonpolar Metabolites Associated With Relapsing Disease in Patients With Resected Oral Cavity Squamous Cell Carcinoma A, Volcano plot illustrating polar metabolites in patients with and without relapse. Metabolites are categorized based on fold changes and adjusted *P* values. Upregulated metabolites (fold change greater than 1.1), downregulated metabolites (fold change less than 0.909), and nonsignificant changes are highlighted. B, Volcano plot of nonpolar metabolites using the same criteria as polar metabolites to identify significant changes between relapsed and nonrelapsed patients. The horizontal dashed line represents the significance threshold (*P* = .05), and the vertical dashed lines represent the fold change thresholds (0.909 and 1.1 on the log_2_ scale).

Initial metabolomic profiling was performed using principal component analysis and partial least-squares discriminant analysis to examine serum metabolite patterns. Detailed results, including principal component analysis and partial least-squares discriminant analysis score plots, are available in eFigure 2 in Supplement 1. Subsequent analysis identified 56 differentially expressed metabolites (36 polar and 20 nonpolar) between patients with OCSCC with and without relapsing disease meeting the statistical significance threshold (*P* < .05) and FC criteria (>1.1 or <0.909). These differences are visualized in volcano plots for both polar and nonpolar metabolites in Figure 1. Further refinement using the XGBoost machine learning algorithm identified 36 key metabolites (20 polar and 16 nonpolar), which underwent subsequent Kaplan-Meier analysis to evaluate their association with DFS.

### Development of the MetaboScore

Optimal cutoff values were determined for each of the 36 key metabolites, with corresponding term plots displayed in eFigure 3 in Supplement 1. Through Kaplan-Meier survival analysis, 19 metabolites demonstrated statistical significance and were incorporated into the MetaboScore, yielding a potential score range of 0 to 19. DFS Kaplan-Meier curves for each scoring metabolite are shown in eFigure 4 in Supplement 1 with metabolite annotations provided in Table 2. Analysis of survival outcomes and MetaboScore distribution patterns identified an optimal threshold of 11, distinguishing patients with high MetaboScores (11 or greater) from those with low MetaboScores (less than 11).

**Table 2.** Metabolites Included in the MetaboScore

Mass to charge ratio	Polarity	Ionization mode	Annotation^a^	Tandem mass spectrometry data available	Direction^b^
120.96607	Polar	Positive	Orthophosphate [M+Na]	No	Low
194.95808	Polar	Negative	Unknown	No	Low
221.08308	Nonpolar	Negative	5,6-Dimethoxy-1-indanecarboxylic acid [M-H]	No	High
235.07908	Polar	Positive	4-[2-(3-Cyano-2-pyridinyl)hydrazino]-4-oxobutanoic acid [M+H]	Yes	Low
246.03231	Polar	Positive	2H-Dibenz[b,f]azepin-2-one [M+K]	No	High
273.25720	Nonpolar	Positive	ent-cassa-12,15-diene [M+H]	No	High
280.16196	Nonpolar	Positive	9-Isobutyl-6-(isobutylsulfanyl)-9H-purin-2-amine [M+H]	No	High
293.23205	Nonpolar	Negative	Diglyceride (34:4/0:0) [M-2H]	No	High
338.78477	Polar	Positive	Unknown	No	Low
339.07152	Polar	Positive	1-(5′-Phosphoribosyl)-5-amino-4-imidazolecarboxamide [M+H]	No	Low
387.38078	Nonpolar	Positive	2,4,22-Docosanetriol, 3,5-dimethyl- [M+H]	No	High
407.29803	Polar	Positive	Sannamycin A; istamycin B [M+NH4]	No	High
429.31834	Polar	Positive	L-Leucyl-L-isoleucylglycyl-L-lysinamide [M+H]	Yes	Low
553.98727	Nonpolar	Negative	Unknown	No	High
580.36434	Nonpolar	Negative	Phosphatidylserine (22:0/0:0) [M-H]	No	Low
653.58294	Nonpolar	Positive	Hydroxyphthioceranic acid (C40) [M+2Na-H]	No	High
655.59896	Nonpolar	Positive	FAHFA (22:3-(O-22:0)); FAHFA (20:3-(O-24:0)); FAHFA (24:3-(O-20:0)) [M+H-H_2_O]	No	High
680.59752	Nonpolar	Positive	5-(Octadecyloxy)-3-[(octadecyloxy)carbonyl]-5-oxopentanoate [M+H]	Yes	High
839.76655	Nonpolar	Positive	Triglyceride (34:5/o-18:0) [M+H]	No	High

Abbreviation: FAHFA, fatty acid-hydroxy fatty acid.

^a^ [M+H], [M-H], [M+Na], [M+K], [M+NH_4_], [M+2Na-H], and [M+H-H_2_O] are types of ion adducts detected in mass spectrometry.

^b^ Direction of metabolite signal alteration associated with improved disease-free survival.

### Patient Characteristics Stratified by MetaboScore

Patients with high MetaboScores ≥11 or greater) demonstrated different clinicopathological features compared with those with lower scores (less than 11). Specifically, high-scoring patients were characterized by a higher proportion of advanced primary tumors (pT3-pT4: 76 of 79 [96.2%] vs 129 of 149 [86.6%]; difference, 9.6 percentage points [pp]; 95% CI, 2.7-16.5) and were more likely to have close surgical margins (4 mm or smaller: 32 [40.5%] vs 33 [22.1%]; difference, 18.4 pp; 95% CI, 5.6-31.1) than patients with low MetaboScores. The high MetaboScore group was also associated with aggressive disease characteristics, including a higher proportion of pN3b nodal involvement (31 [39.2%] vs 41 [27.5%]; difference, 11.7 pp; 95% CI, 1.2-24.7), pathological stage IV disease (70 [88.6%] vs 115 [77.2%]; difference, 11.4 pp; 95% CI, 1.7-21.1), and tumor depth of 10 mm or greater (73 [92.4%] vs 123 [82.6%]; difference, 9.8 pp; 95% CI, 1.4-18.3) (Table 1).

### Relapse Patterns and Survival Outcomes Stratified by MetaboScore

**Figure 2.**
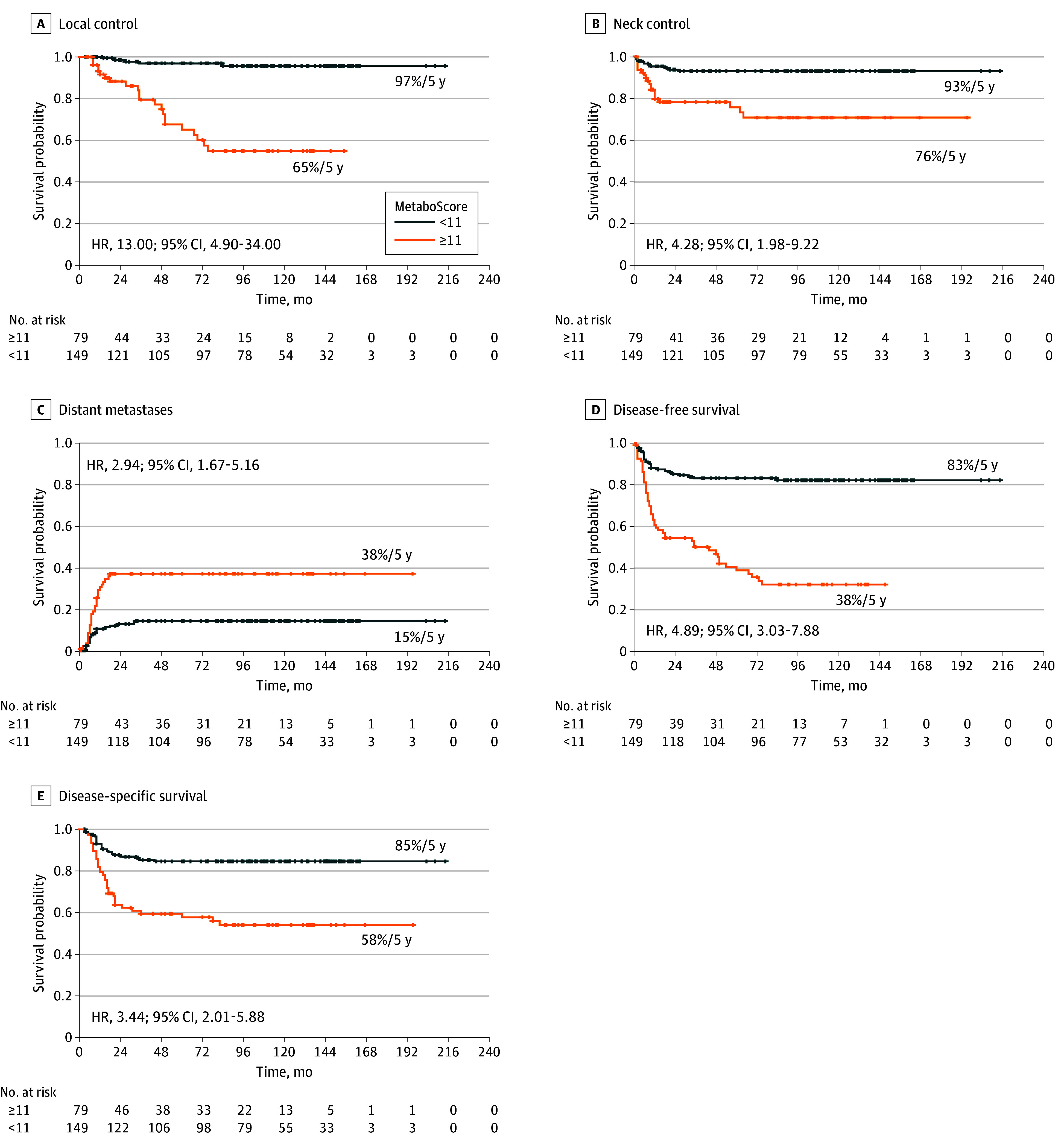
Kaplan-Meier Survival Curves Stratified by MetaboScore in Patients With Resected Oral Cavity Squamous Cell Carcinoma Local control (A), neck control (B), and distant metastases rates (C) stratified by MetaboScore (less than 11 vs 11 or greater). Disease-free survival (D) and disease-specific survival (E) stratified by MetaboScore. HR indicates hazard ratio.

At the 5-year mark, the local control rate was 96.8% (95% CI, 93.7-99.9) in patients with MetaboScores less than 11 compared with 65.0% (95% CI, 52.7-80.2) in those who scored 11 or more (difference, 31.8 pp; 95% CI, 17.7-45.7) (Figure 2A). Similarly, neck control rates were higher in the low MetaboScore group (93.0%; 95% CI, 88.8-97.3) than in patients with scores of 11 or greater (75.6%; 95% CI, 65.9-86.8; difference, 17.3 pp; 95% CI, 6.1-28.5) (Figure 2B). The distant metastases rate was recorded at 14.6% (95% CI, 8.6-20.2) for patients with a MetaboScore less than 11, whereas it was substantially higher at 37.4% (95% CI, 25.6-47.3) for those scoring 11 or greater (difference, 22.8 pp; 95% CI, 10.5-35.0) (Figure 2C). Correspondingly, the 5-year DFS rate was 83% (95% CI, 77-89) in the group with a MetaboScore below 11 compared with 38% (95% CI, 28-51) in those with scores of 11 or greater (difference, 45 pp; 95% CI, 32-57) (Figure 2D). DSS followed a similar pattern, with rates of 85% (95% CI, 79-91) and 58% (95% CI, 48-70) for the less than 11 and 11 or greater groups, respectively (difference, 27 pp; 95% CI, 14-40) (Figure 2E). When stratified by *AJCC Staging Manual* stage, the MetaboScore still showed a strong association with reduced survival outcomes. In patients with stage IV disease (n = 185), scores of 11 or greater were associated with poorer 5-year DFS (33% [95% CI, 32-47] vs 79% [95% CI, 72-87]; difference, 46 pp; 95% CI, 32-60) and DSS (54% [95% CI, 43-67] vs 82% [95% CI, 75-89]; difference, 28 pp; 95% CI, 14-42) compared with scores less than 11. A comparable pattern emerged in patients with stage III disease (n = 43), although the limited sample size and low event count constrained statistical power (eFigure 5 in Supplement 1).

### Univariable and Multivariable Analyses for 5-Year Outcomes

The results of univariable analyses are presented in the eTable in Supplement 1. A MetaboScore of 11 or greater emerged as a robust independent predictor across all study end points in multivariable analysis (Table 3). Specifically, a high MetaboScore demonstrated strong associations with local control (HR, 14.84; 95% CI, 5.45-40.40), neck control (HR, 4.22; 95% CI, 1.94-9.15), distant metastases (HR, 2.53; 95% CI, 1.42-4.50), DFS (HR, 4.40; 95% CI, 2.71-7.15), and DSS (HR, 3.04; 95% CI, 1.76-5.25). Lymphatic invasion consistently appeared as an independent prognostic factor for all 5 outcomes. Additional independent predictors included pN3b status and poor tumor differentiation for distant metastases, pN3b status for DFS, and both pN3b status and vascular invasion for DSS. Notably, while both MetaboScore of 11 or greater and lymphatic invasion maintained independent prognostic significance across all outcomes, the MetaboScore identified a larger high-risk population (n = 79) compared with lymphatic invasion (n = 13).

**Table 3.** Multivariable Analyses of Risk Factors for 5-Year Local Control, Neck Control, Distant Metastases, Disease-Free Survival, and Disease-Specific Survival in Patients With Resected Oral Cavity Squamous Cell Carcinoma

Risk factor^a^	Total, No.	HR (95% CI)				
		Control		Distant metastases	Survival	
		Local	Neck		Disease free	Disease specific
MetaboScore						
≥11	79	14.84 (5.45-40.40)	4.22 (1.94-9.15)	2.53 (1.42-4.50)	4.40 (2.71-7.15)	3.04 (1.76-5.25)
<11	149	1 [Reference]	1 [Reference]	1 [Reference]	1 [Reference]	1 [Reference]
Lymphatic invasion						
Yes	13	9.09 (1.88-43.94)	4.28 (1.49-12.26)	5.45 (2.53-11.74)	4.45 (2.20-9.03)	4.50 (2.02-10.03)
No	215	1 [Reference]	1 [Reference]	1 [Reference]	1 [Reference]	1 [Reference]
pN3b						
Yes	72	NA	NA	4.35 (2.33-9.10)	2.88 (1.80-4.61)	3.90 (2.20-6.91)
No	156	NA	NA	1 [Reference]	1 [Reference]	1 [Reference]
Tumor differentiation						
Poor	36	NA	NA	2.05 (1.09-3.86)	NA	NA
Well to moderate	192	NA	NA	1 [Reference]	NA	NA
Vascular invasion						
Yes	15	NA	NA	NA	NA	3.01 (1.42-6.35)
No	213	NA	NA	NA	NA	1 [Reference]

Abbreviations: HR, hazard ratio; NA, not applicable.

^a^ All 17 factors identified in univariable analysis were entered into the multivariable model; only significant risk factors are presented here.

### LASSO Analysis

To further validate the stability of our stepwise-selected model, we performed a LASSO-penalized Cox regression using the same covariates. At the optimal penalty (λ with the minimum deviance), 6 variables remained in the model, including MetaboScore of 11 or greater (with a negative coefficient for MetaboScore less than 11), whereas at λ 1 SE above the minimum, only 2 variables—pN3 disease and MetaboScore less than 11—retained nonzero coefficients. These findings were closely in line with our stepwise Cox results, underscoring the independent prognostic significance of the MetaboScore. Notably, the consistent retention of MetaboScore less than 11 at both penalty thresholds reaffirms its strong prognostic value for DFS in patients with OCSCC.

### Prognostic Value of the MetaboScore in Patients With pN3b Disease

According to the *AJCC Staging Manual, eighth edition*, the presence of extranodal extension in patients with pN2 disease (*AJCC Staging Manual, seventh edition*) warrants disease reclassification as pN3b. Given the critical prognostic significance of pN3b status in the current AJCC staging system, subgroup analyses were performed to evaluate whether the MetaboScore could provide prognostic information beyond this established staging parameter. Among pN3b-positive patients (n = 72), those with a MetaboScore of 11 or greater demonstrated worse outcomes, with 5-year DFS rates of 20% (95% CI, 9-43) compared with 60% (95% CI, 47-77) in patients scoring less than 11 (difference, 40 pp; 95% CI, 19-62). DSS showed similar disparities, with rates of 28% (95% CI, 15-52) and 62% (95% CI, 49-79) for high and low MetaboScore groups, respectively (difference, 34 pp; 95% CI, 11-58) (eFigure 6A-B in Supplement 1). In pN3b-negative patients (n = 156), a high MetaboScore (11 or greater) was also strongly associated with inferior outcomes. Five-year DFS rates were 50% (95% CI, 37-68) vs 91% (95% CI, 86-97) for patients with high and low MetaboScores, respectively (difference, 41 pp; 95% CI, 25-57), whereas the corresponding DSS rates were 76% (95% CI, 65-90) vs 93% (95% CI, 88-98), respectively (difference, 17 pp; 95% CI, 4-30) (eFigure 6C-D in Supplement 1).

## Discussion

Unlike prior metabolomic studies in OCSCC that primarily analyzed saliva or assessed overall survival outcomes, our investigation assessed the association of serum metabolomics with disease recurrence in patients with OCSCC. Importantly, our dataset was continuously updated through a centralized registry, allowing for seamless incorporation of the *AJCC Staging Manual, eighth edition *staging and minimizing the risk of misclassification. By focusing on DFS patterns, we identified a panel of 19 metabolites that distinguished between patients who experienced disease relapse and those who remained disease free throughout follow-up. These findings led to the development of the MetaboScore, a novel prognostic tool that effectively stratified patients based on their risk of disease recurrence. Notably, the MetaboScore demonstrated independent predictive value for multiple relapse patterns, including local recurrence, neck recurrence, and distant metastases. Furthermore, analysis of patient characteristics showed that high MetaboScores (11 or greater) was correlated with adverse pathological features, such as advanced tumor stage and compromised surgical margins. These associations suggest that the MetaboScore reflects fundamental aspects of OCSCC biology that contribute to disease aggressiveness and increased relapse probability, explaining its consistent prognostic capability across different recurrence patterns.

Furthermore, subgroup analyses revealed that the MetaboScore offered prognostic information beyond pN3b status. Patients with OCSCC with pN3b disease typically have poor neck control and are prone to distant metastases.^28^ While pN3b was a significant risk factor for neck control in univariable analysis (eTable in Supplement 1), it was not retained as an independent predictor in the multivariable model, likely due to being overruled by the MetaboScore. In patients without pN3b disease, a high MetaboScore identified those at an increased likelihood of relapse, even if their pathological stage alone would have classified them as low risk. These findings demonstrate that the MetaboScore provides additional prognostic stratification beyond current AJCC staging criteria, effectively identifying high-risk patients within both pN3b-positive and pN3b-negative groups. In addition, the consistent performance of the MetaboScore across multiple end points underscores its potential value in enhancing clinical management strategies for patients with OCSCC.

From an analytical standpoint, the MetaboScore includes both endogenous and exogenous metabolites (Table 2), thereby capturing the complex mechanisms underlying OCSCC recurrence. At its core, orthophosphate serves as a fundamental metabolic cornerstone, orchestrating multiple cellular processes. The higher DFS observed in the low-orthophosphate group aligns with our previous genetic study, in which Harvey rat sarcoma viral oncogene homolog (HRAS)—a key upstream regulator of rat sarcoma (Ras)–rapidly accelerated fibrosarcoma (Raf)–mitogen-activated protein kinase kinase (MEK)–extracellular signal-regulated kinase (ERK) phosphorylation cascades and phosphate-utilizing metabolic pathways—was identified as a significant predictor of 5-year DFS in OCSCC.^29,30,31^ Oncogenic HRAS promotes metabolic reprogramming by enhancing glycolytic flux and increasing ATP production, which correlates with orthophosphate consumption in tumor cells.^32^ Another key component of the score, 1-(5′-phosphoribosyl)-5-amino-4-imidazolecarboxamide, functions as a crucial purine biosynthesis intermediate and AMP-activated protein kinase pathway activator, exhibiting tumor-suppressive properties through anabolic process inhibition and autophagy promotion.^33^ The score further incorporates diverse biomolecules essential for cellular function, including the amino acid–derived metabolite L-Leucyl-L-isoleucylglycyl-L-lysinamide, alongside 3 specific lipid components: diacylglycerol (34:4/0:0), phosphatidylserine (22:0/0:0), and triglyceride (34:5/o-18:0). These metabolites collectively modulate membrane architecture and signal transduction pathways critical for cellular proliferation, migration, and apoptosis.^13,34,35^ Notably, similar metabolomic alterations have also been reported in other head and neck squamous cell carcinoma subtypes,^36,37,38,39^ suggesting the potential broader applicability of the MetaboScore beyond OCSCC. Conversely, the exogenous components of the MetaboScore provide insights into potential environmental influences and therapeutic interventions in OCSCC. The aminoglycoside antibiotics sannamycin A and istamycin B may serve as indicators of underlying infection states. The presence of 2H-Dibenz[b,f]azepin-2-one may reflect the complex interplay of psychological and somatic symptoms in OCSCC. The score also included complex fatty acid derivatives, including fatty acid esters of hydroxy fatty acids and 5-(octadecyloxy)-3-[(octadecyloxy)carbonyl]-5-oxopentanoate, which may regulate innate and adaptive immune responses.^40^ The additional exogenous metabolites included in the score likely originate from diverse sources, underscoring the multifaceted nature of metabolic influences in driving OCSCC recurrences.

### Limitations

Despite promising results, our study has several limitations. First, the retrospective design and single-center setting may limit the broader applicability of our conclusions, despite the samples being collected according to a preset protocol through the institutional tissue bank. The prevalent betel quid–related etiology and predominance of male patients with advanced-stage disease in our local population can also restrict generalizability to other settings. In Taiwan, betel quid chewing prevalence is considerably higher in male individuals than in female individuals (20.9% vs 1.2%, respectively),^41^ which may contribute to the marked difference in OCSCC incidence between men (27.08 per 1 000 000) and women (2.76 per 1 000 000).^42^ Second, information on comorbidities, nutritional status, body mass index, diabetes, and concomitant medications, particularly those affecting metabolism, was not systematically collected in our institutional registry, representing a potential confounding factor that should be addressed in future prospective validation studies. Third, although robust statistical analyses (univariable, multivariable, and LASSO regression) were used, the multistep selection process used to develop the MetaboScore could raise concerns about potential overfitting, necessitating external validation. Fourth, the biological significance of identified metabolites requires further mechanistic exploration. Finally, integrating metabolomics with genomic and proteomic data could further improve the prognostic accuracy.

For future clinical studies, implementing a serum-based metabolite panel into routine clinical practice presents several barriers, including cost constraints, the technical requirements of mass spectrometry, and prolonged processing times. Accordingly, routine metabolomic workflows may extend over several weeks, from initial sample collection to the availability of results. Nevertheless, the development of targeted mass spectrometry–based assays focusing on the 19 MetaboScore metabolites has the potential to shorten turnaround to a few days, comparable with current pathological processing timelines. Prospective and multicenter validation of the MetaboScore is essential to confirm its clinical benefit and evaluate whether tailored therapeutic strategies based on metabolic risk stratification can ultimately improve patient outcomes.

## Conclusions

In this study, we developed and validated a novel serum-based metabolomic scoring system, the MetaboScore, comprising 19 metabolites to predict recurrence in patients with resected OCSCC. The MetaboScore robustly stratified patients by recurrence risk, independently predicting local, regional, and distant failures, as well as DFS and DSS, including in high-risk patients with pN3b disease. Pending external validation, the MetaboScore could enhance personalized therapeutic decision-making and improve clinical outcomes in the management of OCSCC.

## Notes

Supplement 1.
eMethods.
**eFigure 1.** Patient Selection Process and Disease Recurrence Patterns **eFigure 2.** PCA and PLS-DA of Serum Metabolites **eFigure 3.** Penalized Spline Term Plots **eFigure 4.** DFS Kaplan-Meier Curves by Metabolite Intensity **eFigure 5.** AJCC Stage Specific DFS and DSS by MetaboScore **eFigure 6.** pN3b Specific DFS and DSS by MetaboScore **eTable.** Univariable Analyses

Supplement 2. Data Sharing Statement

## References

[1] Cunha ARD, Compton K, Xu R, ; GBD 2019 Lip, Oral, and Pharyngeal Cancer Collaborators. The global, regional, and national burden of adult lip, oral, and pharyngeal cancer in 204 countries and territories: a systematic analysis for the Global Burden of Disease Study 2019. JAMA Oncol. 2023;9(10):1401-1416. doi:10.1001/jamaoncol.2023.2960 37676656 PMC10485745

[2] Bray F, Laversanne M, Sung H, . Global cancer statistics 2022: GLOBOCAN estimates of incidence and mortality worldwide for 36 cancers in 185 countries. CA Cancer J Clin. 2024;74(3):229-263. doi:10.3322/caac.21834 38572751

[3] Mody MD, Rocco JW, Yom SS, Haddad RI, Saba NF. Head and neck cancer. Lancet. 2021;398(10318):2289-2299. doi:10.1016/S0140-6736(21)01550-6 34562395

[4] Kerawala C, Roques T, Jeannon JP, Bisase B. Oral cavity and lip cancer: United Kingdom National Multidisciplinary Guidelines. J Laryngol Otol. 2016;130(S2):S83-S89. doi:10.1017/S0022215116000499 27841120 PMC4873943

[5] National Comprehensive Cancer Network. Head and Neck Cancers (Version 5.2025). Accessed September 1, 2025. https://www.nccn.org/professionals/physician_gls/pdf/head-and-neck.pdf

[6] Lin CY, Fan KH, Lee LY, . Precision adjuvant therapy based on detailed pathologic risk factors for resected oral cavity squamous cell carcinoma: long-term outcome comparison of CGMH and NCCN guidelines. Int J Radiat Oncol Biol Phys. 2020;106(5):916-925. doi:10.1016/j.ijrobp.2019.08.058 31499138

[7] Struckmeier AK, Buchbender M, Lutz R, Kesting M. Improved recurrence rates and progression-free survival in primarily surgically treated oral squamous cell carcinoma—results from a German tertiary medical center. Clin Oral Investig. 2024;28(5):262. doi:10.1007/s00784-024-05644-z 38642146 PMC11032275

[8] Liao CT, Kang CJ, Lee LY, . Association between multidisciplinary team care approach and survival rates in patients with oral cavity squamous cell carcinoma. Head Neck. 2016;38(suppl 1):E1544-E1553. doi:10.1002/hed.24276 26890807

[9] Holmes E, Wilson ID, Nicholson JK. Metabolic phenotyping in health and disease. Cell. 2008;134(5):714-717. doi:10.1016/j.cell.2008.08.026 18775301

[10] Hanahan D, Weinberg RA. Hallmarks of cancer: the next generation. Cell. 2011;144(5):646-674. doi:10.1016/j.cell.2011.02.013 21376230

[11] Yang X, Song X, Zhang X, . In situ DESI-MSI lipidomic profiles of mucosal margin of oral squamous cell carcinoma. EBioMedicine. 2021;70:103529. doi:10.1016/j.ebiom.2021.103529 34391097 PMC8374374

[12] Yang XH, Zhang XX, Jing Y, . Amino acids signatures of distance-related surgical margins of oral squamous cell carcinoma. EBioMedicine. 2019;48:81-91. doi:10.1016/j.ebiom.2019.10.005 31631041 PMC6838421

[13] Polachini GM, de Castro TB, Smarra LFS, . Plasma metabolomics of oral squamous cell carcinomas based on NMR and MS approaches provides biomarker identification and survival prediction. Sci Rep. 2023;13(1):8588. doi:10.1038/s41598-023-34808-2 37237049 PMC10220089

[14] Song X, Yang X, Narayanan R, . Oral squamous cell carcinoma diagnosed from saliva metabolic profiling. Proc Natl Acad Sci U S A. 2020;117(28):16167-16173. doi:10.1073/pnas.2001395117 32601197 PMC7368296

[15] Yao Z, An W, Tuerdi M, Zhao J. Identification of novel prognostic indicators for oral squamous cell carcinoma based on proteomics and metabolomics. Transl Oncol. 2023;33:101672. doi:10.1016/j.tranon.2023.101672 37084685 PMC10172993

[16] Lee LY, Lin CY, Cheng NM, . Poor tumor differentiation is an independent adverse prognostic variable in patients with locally advanced oral cavity cancer—comparison with pathological risk factors according to the NCCN guidelines. Cancer Med. 2021;10(19):6627-6641. doi:10.1002/cam4.4195 34533269 PMC8495291

[17] Emwas AH, Zacharias HU, Alborghetti MR, . Recommendations for sample selection, collection and preparation for NMR-based metabolomics studies of blood. Metabolomics. 2025;21(3):66. doi:10.1007/s11306-025-02259-7 40348843 PMC12065766

[18] Simundic AM, Cornes M, Grankvist K, Lippi G, Nybo M. Standardization of collection requirements for fasting samples: for the Working Group on Preanalytical Phase (WG-PA) of the European Federation of Clinical Chemistry and Laboratory Medicine (EFLM). Clin Chim Acta. 2014;432:33-37. doi:10.1016/j.cca.2013.11.008 24269503

[19] Chien CY, Wang CP, Lee LY, . Indications for elective neck dissection in cT1N0M0 oral cavity cancer according to the AJCC eight edition: a nationwide study. Oral Oncol. 2023;140:106366. doi:10.1016/j.oraloncology.2023.106366 36965411

[20] Kang CJ, Wen YW, Lee SR, . Towards an improved pathological node classification for prognostic stratification of patients with oral cavity squamous cell carcinoma: results from a nationwide registry study. Front Oncol. 2022;12:910158. doi:10.3389/fonc.2022.910158 35837108 PMC9273780

[21] Diggle PJ, Liang KY, Zeger SL. Analysis of Longitudinal Data. Oxford University Press; 1994.

[22] Draper NR, Smith H. Applied Regression Analysis. John Wiley & Sons; 1998. doi:10.1002/9781118625590

[23] Andersen PK, Gill RD. Cox’s regression model for counting processes: a large sample study. Ann Stat. 1982;10(4):1100-1120. doi:10.1214/aos/1176345976

[24] Therneau TM, Grambsch PM. Modeling Survival Data: Extending the Cox Model. Springer Science & Business Media; 2000. doi:10.1007/978-1-4757-3294-8

[25] Friedman J, Hastie T, Tibshirani R. Regularization paths for generalized linear models via coordinate descent. J Stat Softw. 2010;33(1):1-22. doi:10.18637/jss.v033.i01 20808728 PMC2929880

[26] Tibshirani R. The lasso method for variable selection in the Cox model. Stat Med. 1997;16(4):385-395. doi:10.1002/(SICI)1097-0258(19970228)16:4<385::AID-SIM380>3.0.CO;2-3 9044528

[27] Tibshirani R. Regression shrinkage and selection via the lasso. J R Stat Soc B. 2018;58(1):267-288. doi:10.1111/j.2517-6161.1996.tb02080.x

[28] Liao CT, Lee LY, Hsueh C, . Pathological risk factors stratification in pN3b oral cavity squamous cell carcinoma: focus on the number of positive nodes and extranodal extension. Oral Oncol. 2018;86:188-194. doi:10.1016/j.oraloncology.2018.09.021 30409299

[29] Fan WL, Yang LY, Hsieh JC, Lin TC, Lu MJ, Liao CT. Prognostic genetic biomarkers based on oncogenic signaling pathways for outcome prediction in patients with oral cavity squamous cell carcinoma. Cancers (Basel). 2021;13(11):2709. doi:10.3390/cancers13112709 34070941 PMC8199274

[30] Liao CT, Hsieh CH, Fan WL, . A combined analysis of maximum standardized uptake value on FDG-PET, genetic markers, and clinicopathological risk factors in the prognostic stratification of patients with resected oral cavity squamous cell carcinoma. Eur J Nucl Med Mol Imaging. 2020;47(1):84-93. doi:10.1007/s00259-019-04453-x 31388722

[31] Cancer Genome Atlas N; Cancer Genome Atlas Network. Comprehensive genomic characterization of head and neck squamous cell carcinomas. Nature. 2015;517(7536):576-582. doi:10.1038/nature14129 25631445 PMC4311405

[32] Wu X, Adame-Garcia SR, Koshizuka K, . Oncogenic HRAS induces metformin resistance in head and neck cancer by promoting glycolytic metabolism. Cancer Prev Res (Phila). 2024;17(12):571-583. doi:10.1158/1940-6207.CAPR-24-0124 39463147 PMC11969736

[33] Knobloch TJ, Ryan NM, Bruschweiler-Li L, . Metabolic regulation of glycolysis and AMP activated protein kinase pathways during black raspberry-mediated oral cancer chemoprevention. Metabolites. 2019;9(7):140. doi:10.3390/metabo9070140 31336728 PMC6680978

[34] Vitório JG, Duarte-Andrade FF, Dos Santos Fontes Pereira T, . Metabolic landscape of oral squamous cell carcinoma. Metabolomics. 2020;16(10):105. doi:10.1007/s11306-020-01727-6 33000429

[35] Hsu CW, Chen YT, Hsieh YJ, . Integrated analyses utilizing metabolomics and transcriptomics reveal perturbation of the polyamine pathway in oral cavity squamous cell carcinoma. Anal Chim Acta. 2019;1050:113-122. doi:10.1016/j.aca.2018.10.070 30661578

[36] Ohashi T, Terazawa K, Shibata H, Inoue N, Ogawa T. Metabolic profiling analysis of head and neck squamous cell carcinoma. Oral Dis. 2024;30(2):342-352. doi:10.1111/odi.14432 36349421

[37] Somashekar BS, Kamarajan P, Danciu T, . Magic angle spinning NMR-based metabolic profiling of head and neck squamous cell carcinoma tissues. J Proteome Res. 2011;10(11):5232-5241. doi:10.1021/pr200800w 21961579 PMC3208743

[38] Shin JM, Kamarajan P, Fenno JC, Rickard AH, Kapila YL. Metabolomics of head and neck cancer: a mini-review. Front Physiol. 2016;7:526. doi:10.3389/fphys.2016.00526 27877135 PMC5099236

[39] Tang T, Zhou Z, Chen M, . Plasma metabolic profiles-based prediction of induction chemotherapy efficacy in nasopharyngeal carcinoma: results of a bidirectional clinical trial. Clin Cancer Res. 2024;30(14):2925-2936. doi:10.1158/1078-0432.CCR-23-3608 38713248 PMC11247322

[40] Yore MM, Syed I, Moraes-Vieira PM, . Discovery of a class of endogenous mammalian lipids with anti-diabetic and anti-inflammatory effects. Cell. 2014;159(2):318-332. doi:10.1016/j.cell.2014.09.035 25303528 PMC4260972

[41] Yang YH, Chen HR, Tseng CH, Shieh TY. Prevalence rates of areca/betel quid chewing in counties of Taiwan. Taiwan J Oral Med Health Sci. 2002;18(1):1-16. doi:10.7059/TJOMHS.200210.0001

[42] Taiwan Cancer Registry. 2007-2022 Annual report. Accessed September 1, 2025. https://www.hpa.gov.tw/Pages/TopicList.aspx?nodeid=269

