# Supplementary material for: Serum Metabolomics for Prognostic Stratification in Resected Advanced-Stage Oral Cavity Cancer
Source: JAMA Otolaryngol Head Neck Surg. 2025 Dec 4;152(2):172–81. doi: 10.1001/jamaoto.2025.4267 (PMC12679420; doi:10.1001/jamaoto.2025.4267)
Supplement: Supplement 1. — eMethods. eFigure 1. Patient Selection Process and Disease Recurrence Patterns eFigure 2. PCA and PLS-DA of Serum Metabolites eFigure 3. Penalized Spline Term Plots eFigure 4. DFS Kaplan-Meier Curves by Metabolite Intensity eFigure 5. AJCC Stage Specific DFS and DSS by MetaboScore eFigure 6. pN3b Specific DFS and DSS by MetaboScore eTable. Univariable Analyses [file jamaotolaryngolheadnecksurg-e254267-s001.pdf]

Supplemental Online Content

Shen EYL, Lee LY, Ng SH, et al. Serum metabolomics for prognostic stratification in resected advanced-stage oral cavity cancer. *JAMA Otolaryngol Head Neck Surg*. Published online DATE. doi:10.1001/jamaoto.2025.4267

eMethods.

eFigure 1. Patient Selection Process and Disease Recurrence Patterns

eFigure 2. PCA and PLS-DA of Serum Metabolites

eFigure 3. Penalized Spline Term Plots

eFigure 4. DFS Kaplan-Meier Curves by Metabolite Intensity

eFigure 5. AJCC Stage Specific DFS and DSS by MetaboScore

eFigure 6. pN3b Specific DFS and DSS by MetaboScore

eTable. Univariable Analyses

This supplementary material has been provided by the authors to give readers additional information about their work.

© 2025 Shen EYL et al. *JAMA Otolaryngology–Head and Neck Surgery*.

eMethods.

Treatment protocols

The therapeutic protocol included surgical intervention with tumor excision targeting a minimum 1-cm safety margin, accompanied by neck dissection when clinically indicated. Post-operative management comprised adjuvant RT or CRT, determined by the presence of pathological risk factors. Risk factors were classified according to the NCCN guidelines until 2008; thereafter, the CGMH guidelines were adopted.[1]

Radiation therapy was administered at doses of 6000 to 6600 cGy, delivered across 30–33 fractions. Concurrent chemotherapy protocols utilized cisplatin-based regimens, administered either as weekly intravenous doses of 40 mg/m^2, biweekly doses of 50 mg/m^2 combined with daily oral tegafur 800 mg and leucovorin 60 mg, or triweekly doses of 100 mg/m^2.

Follow-up surveillance

After completing treatment, the follow-up protocol consisted of head and neck physical examinations conducted every 1 to 3 months during the first year, every 2 to 4 months in the second year, every 3 to 6 months in the third year, and every 6 to 12 months thereafter. Post-treatment imaging, using MRI and ^{18}F-FDG PET scans, was initially performed at the three-month mark and then annually unless there was a clinical suspicion of tumor recurrence. At each follow-up visit, data on clinical events – including local control, neck control, distant metastasis, disease-free survival (DFS), disease-specific survival (DSS), and OS – were updated. Each follow-up assessment prioritized the detection of recurrent disease as the primary surveillance outcome.

© 2025 Shen EYL et al. *JAMA Otolaryngology–Head and Neck Surgery*.

Salvage therapy for disease recurrences

Disease recurrences were confirmed either histologically or through MDT review of imaging findings.[2] Patients in whom local tumor recurrences were deemed resectable underwent radical surgical excision with safety margins. A comprehensive neck dissection encompassing levels I–V was performed for patients presenting with neck nodal recurrences. Decisions regarding the administration of adjuvant RT or CRT were made during MDT meetings. Patients with unresectable recurrences were managed with definitive RT, CRT, or supportive care, as determined by the MDT based on the patient's overall conditions. In cases of distant failures, additional systemic treatment was recommended.

Serum sample processing and mass spectrometry

Following a minimum 6-h fasting period on the day of surgery, venous blood specimens were collected and centrifuged to obtain serum, which was immediately cryopreserved at -80°C. All serum samples used in this study were collected through a prospectively registered institutional tissue bank, which standardized pre-analytical procedures to minimize variability. Rigorous protocols for cryopreservation and sample traceability were followed to reduce biases typically associated with retrospective collections.

Sample processing began with thawing specimens on ice, followed by the addition of 240 µL extraction buffer (comprising methanol, acetonitrile, and double-distilled water in a 5:3:2 volumetric ratio, maintained at -20°C) to 10 µL serum aliquots. The mixture underwent vortexing and centrifugation at 12,000 g for 30 min at 4°C. A 170 µL supernatant volume was subsequently transferred to vials and stored at -20°C pending flow-injection analysis (FIA) using a Q-Exactive Plus Orbitrap mass spectrometer (Thermo Fisher Scientific, Waltham, MA, USA). Analytic parameters included a 3-min injection duration with 10 µL

© 2025 Shen EYL et al. *JAMA Otolaryngology–Head and Neck Surgery*.

sample volume, 50 µL/min flow rate, and a mobile phase consisting of 0.1% formic acid in 5% acetonitrile. Samples were electrosprayed at 4 kV and 325°C, with mass scanning performed across an m/z range of 60–900 at 140,000 resolution. Sequential positive and negative ionization modes were employed to obtain comprehensive metabolic profiles. Detailed mass spectrometry quality assurance and quality control (QA/QC) protocols and data preprocessing methods are reported in the Supplement below.

Mass spectrometry quality assurance and quality control

To ensure analytical quality and reliability, we implemented a comprehensive control strategy incorporating multiple reference samples throughout our analysis. The control framework consisted of three distinct sample types. First, we created a testing matrix sample by pooling aliquots from each experimental sample, providing an averaged representation of the complete sample set. Second, we employed a quality control (QC) matrix sample, consisting of well-characterized human serum (Sigma-Aldrich, S1-M, human serum, normal), which served as a consistent technical replicate across all datasets and enabled monitoring of analytical stability. Finally, we included process blanks, comprising extracted water samples, to detect and monitor potential contamination during the analytical workflow. Instrument variability was assessed by calculating the median relative standard deviation (RSD) for standards added to each sample prior to injection into the mass spectrometer. Bland-Altman plots were utilized to evaluate the agreement between two separate runs for QC.[3] Overall process variability was determined by computing the median RSD for all endogenous metabolites present in 100% of the pooled QC matrix samples. Experimental samples were randomized across platform runs and analyzed continuously over 3 days with minimal interruptions. To address potential blood processing

© 2025 Shen EYL et al. *JAMA Otolaryngology–Head and Neck Surgery*.

and batch variance, QC matrix samples were evenly distributed (every ten testing sera) among the injections to ensure both intra- and inter-batch consistency in the metabolomics analysis. All statistical analyses were conducted using R, version 4.0.3 (R Foundation for Statistical Computing, Vienna, Austria) and associated packages.

Mass spectrometry data processing

The raw mass spectrometry data files were processed using ProteoWizard for conversion and centroiding, and subsequently saved in the mzXML format.[4] Injection zones and mass-to-charge (m/z) bands were identified using the proFIA package (version 1.15.0) for preprocessing mass spectrometry data.[5] The detected bands were aligned to generate a data matrix across samples within the same batch using the MzClust method from the xcms package (version 3.12.0) for high-resolution mass spectrometric spectra analysis.[6] A robust locally estimated scatterplot smoothing (LOESS) model was applied to each set of aligned bands to correct for intra-batch drift during the injection process.[7] Inter-batch differences were addressed by normalizing to the median of the normal baseline for each set of aligned bands (i.e., metabolic features). Metabolic features with more than 30% missing values were excluded. The filtered data matrix was then log2-transformed, centered, and scaled using the means and standard deviations of the normal population for each metabolic feature. Finally, missing values were imputed using the k-nearest neighbor (kNN) algorithm from the impute package (version 1.64.0).

Metabolite annotation

Metabolites were annotated through spectral matching with public databases, including the Human Metabolome Database (HMDB) (RRID: SCR_007712),[8] MassBank of North America

© 2025 Shen EYL et al. *JAMA Otolaryngology–Head and Neck Surgery*.

(MoNA) (RRID: SCR_015536), MassBank (RRID: SCR_015535), METLIN (RRID: SCR_010500),[9] and National Institute of Standards and Technology (NIST) (RRID: SCR_006440). To enhance confidence in metabolite identification, samples were also analyzed using liquid chromatography-tandem mass spectrometry (LC-MS/MS) in data-dependent acquisition (DDA) mode.[10]

Relapse patterns

During a median follow-up period of 81 months (mean: 86 months; standard deviation: 55 months) from surgery, 76 patients developed tumor relapse. The recurrence patterns were distributed as follows: isolated local recurrence (n = 16), isolated nodal recurrence (n = 4), isolated distant metastases (n = 28), combined local and nodal recurrences (n = 6), combined local and metastatic recurrences (n = 3), combined nodal and metastatic recurrences (n = 17), and concurrent local, nodal, and metastatic recurrences (n = 2). The remaining 152 patients remained disease-free throughout follow-up (Figure 1). The 5-year rates were 87.4% for local control, 87.3% for neck control, 22.6% for distant metastases, 67.5% for DFS, and 75.4% for DSS.

Serum metabolomic profiles of patients with and without relapsing disease

In the collected serum samples, we identified a total of 4,469 metabolites (2,881 polar and 1,588 non-polar). After filtering out analytes with missing value ratios exceeding 30%, 4,237 metabolites were retained (2,731 polar and 1,506 non-polar). Serum metabolomic profiles were then compared between OCSCC patients who experienced disease recurrence (n = 76) and those who remained disease-free (n = 152). PCA revealed that the first principal component (PC1) accounted for the largest proportion of variance in both categories (27.4%

© 2025 Shen EYL et al. *JAMA Otolaryngology–Head and Neck Surgery*.

for polar and 18.0% for non-polar metabolites), representing the most significant intergroup difference. PLS-DA demonstrated clear separation between patients with and without disease relapse for polar metabolites, while non-polar metabolites showed substantial overlap. The corresponding PCA and PLS-DA score plots for both polar and non-polar metabolites are presented in eFigure 2 in the Supplement.

References

1. Lin C-Y, Fan K-H, Lee L-Y, et al. Precision Adjuvant Therapy Based on Detailed Pathologic Risk Factors for Resected Oral Cavity Squamous Cell Carcinoma: Long-Term Outcome Comparison of CGMH and NCCN Guidelines. *Int J Radiat Oncol Biol Phys.* 2020;106(5):916-925.
2. Liao CT, Kang CJ, Lee LY, et al. Association between multidisciplinary team care approach and survival rates in patients with oral cavity squamous cell carcinoma. *Head Neck.* 2016;38 Suppl 1:E1544-1553.
3. Giavarina D. Understanding Bland Altman analysis. *Biochem Med (Zagreb).* 2015;25(2):141-151.
4. Kessner D, Chambers M, Burke R, Agus D, Mallick P. ProteoWizard: open source software for rapid proteomics tools development. *Bioinformatics.* 2008;24(21):2534-2536.
5. Delabrière A, Hohenester UM, Colsch B, Junot C, Fenaille F, Thévenot EA. proFIA: a data preprocessing workflow for flow injection analysis coupled to high-resolution mass spectrometry. *Bioinformatics.* 2017;33(23):3767-3775.

© 2025 Shen EYL et al. *JAMA Otolaryngology–Head and Neck Surgery*.

6. Smith CA, Want EJ, O'Maille G, Abagyan R, Siuzdak G. XCMS: Processing Mass Spectrometry Data for Metabolite Profiling Using Nonlinear Peak Alignment, Matching, and Identification. *Anal Chem.* 2006;78(3):779-787.

7. Dunn WB, Broadhurst D, Begley P, et al. Procedures for large-scale metabolic profiling of serum and plasma using gas chromatography and liquid chromatography coupled to mass spectrometry. *Nat Protoc.* 2011;6(7):1060-1083.

8. Wishart DS, Feunang YD, Marcu A, et al. HMDB 4.0: the human metabolome database for 2018. *Nucleic Acids Research.* 2017;46(D1):D608-D617.

9. Smith CA, Maille GO, Want EJ, et al. METLIN. *Therapeutic Drug Monitoring.* 2005;27(6):747-751.

10. Schrimpe-Rutledge AC, Codreanu SG, Sherrod SD, McLean JA. Untargeted Metabolomics Strategies-Challenges and Emerging Directions. *J Am Soc Mass Spectrom.* 2016;27(12):1897-1905.

© 2025 Shen EYL et al. *JAMA Otolaryngology–Head and Neck Surgery*.

eFigure 1. Patient Selection Process and Disease Recurrence Patterns

Patient selection process and disease recurrence patterns. Three patients were excluded because the number of detected features in their serum specimens was less than 10% of the relative standard deviation (RSD) for the total feature count in FIA-MS analysis. Abbreviations: OCSCC, oral cavity squamous cell carcinoma; AJCC, American Joint Committee on Cancer; RSD, relative standard deviation; T, local recurrence; N: neck recurrence; M: distant metastasis.

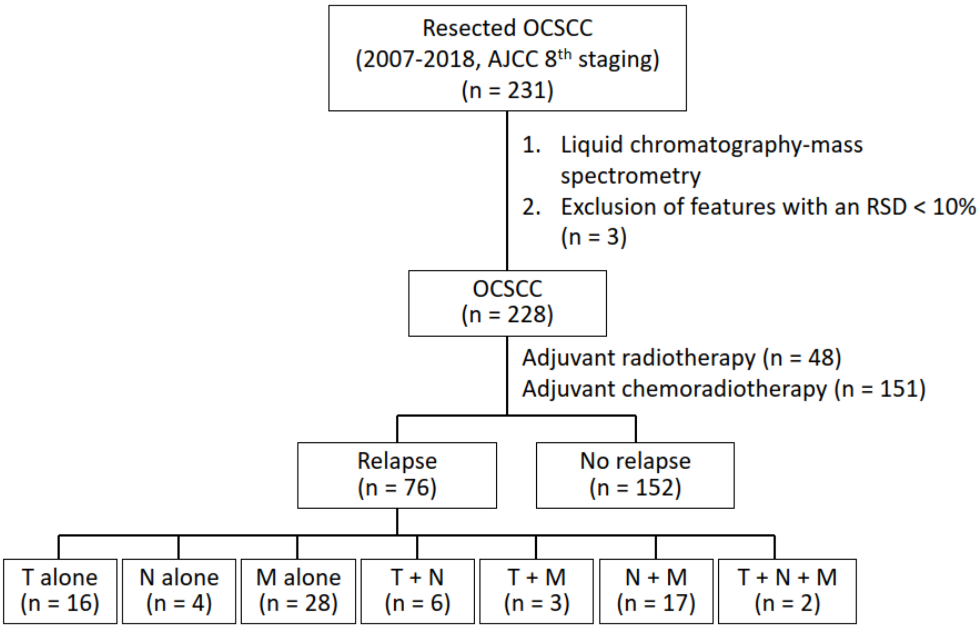

© 2025 Shen EYL et al. *JAMA Otolaryngology–Head and Neck Surgery*.

eFigure 2. PCA and PLS-DA of Serum Metabolites

Principal component analysis (PCA) and partial least squares discriminant analysis (PLS-DA) of serum metabolites. (A, B) PCA score plots for polar (A) and non-polar metabolites (B), showing case (red) and control (green) distributions across the first five principal components with variance percentages indicated. (C, D) PLS-DA score plots for polar (C) and non-polar metabolites (D), illustrating case (red) and control (blue) separation with 95% confidence ellipses. Abbreviation: PC, principal component.

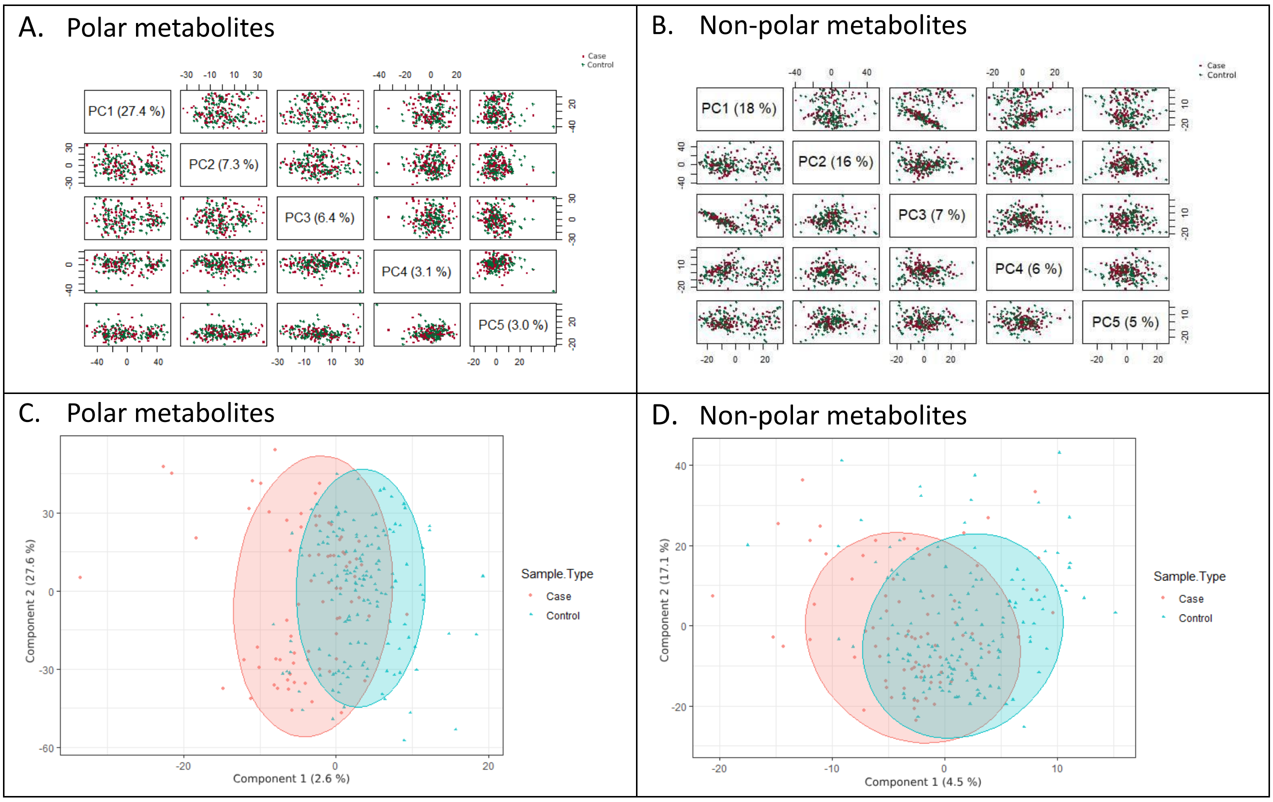

© 2025 Shen EYL et al. *JAMA Otolaryngology–Head and Neck Surgery*.

eFigure 3. Penalized Spline Term Plots

Adjusted hazard ratios for disease-free survival based on metabolite signal intensities. Hazard ratios (HRs) for disease-free survival are modeled against intensities of 36 metabolites using Cox regression with penalized splines. The x-axis represents metabolite intensity, whereas the y-axis displays HRs. The horizontal blue line at HR = 1 represents the point of no effect on relapse risk, indicating a neutral association between metabolite intensity and hazard. Points where the HR approaches 1 suggest potential optimal cutoffs for metabolite intensities. For figures with multiple intersections crossing the HR = 1 line, the cutoff closest to HR = 1 was selected as the most representative intensity value.

© 2025 Shen EYL et al. *JAMA Otolaryngology–Head and Neck Surgery*.

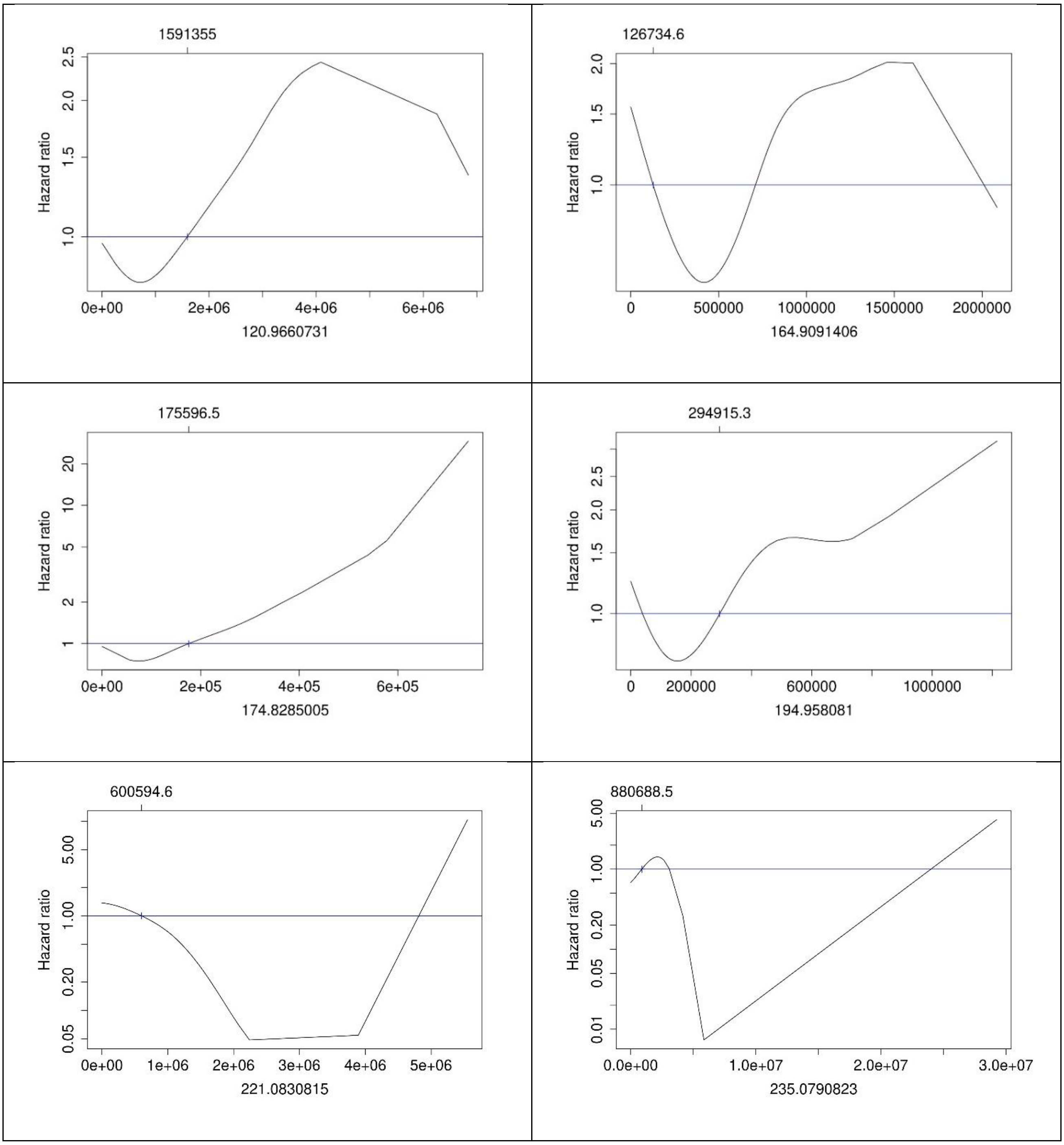

© 2025 Shen EYL et al. *JAMA Otolaryngology–Head and Neck Surgery*.

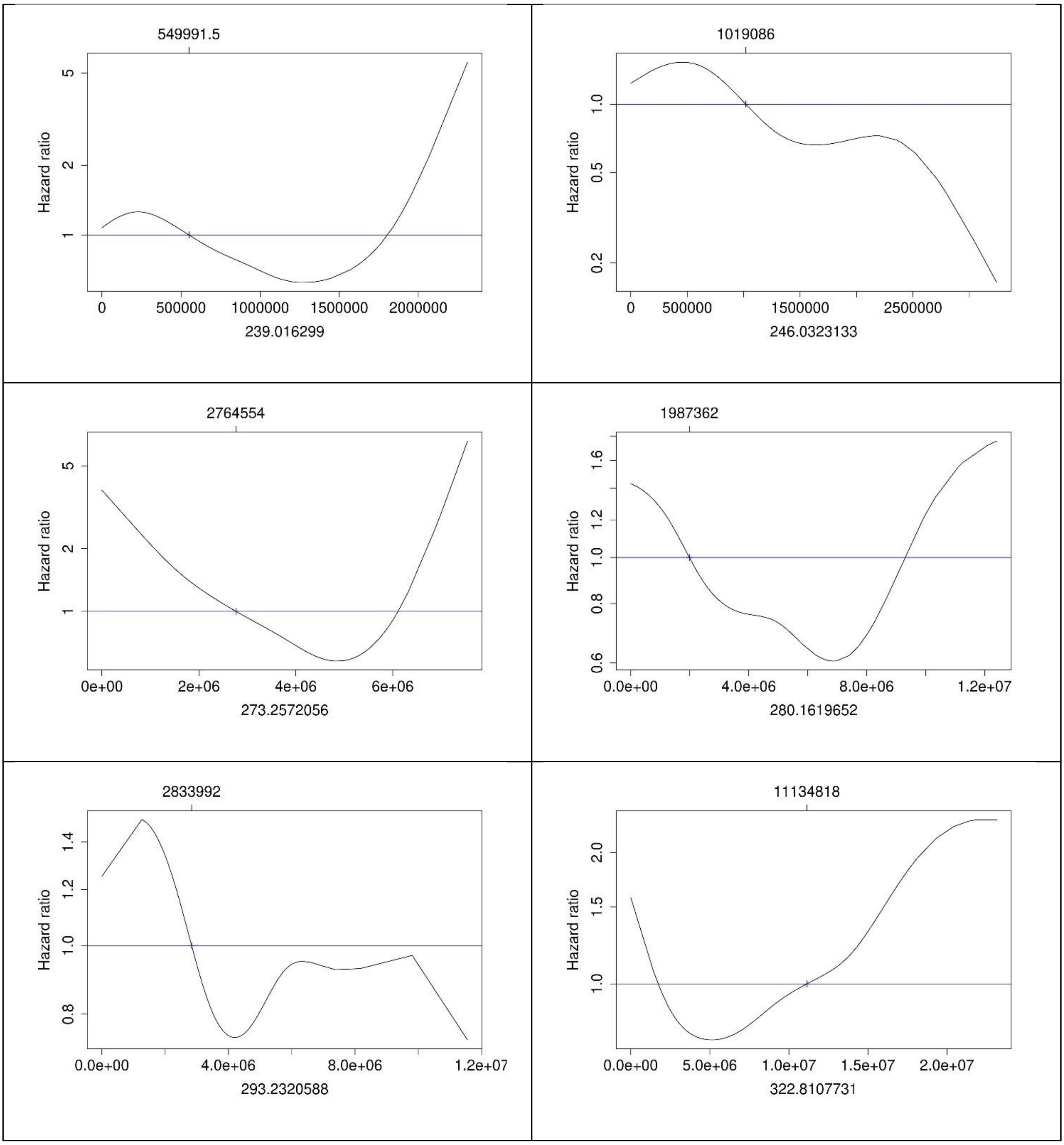

© 2025 Shen EYL et al. *JAMA Otolaryngology–Head and Neck Surgery*.

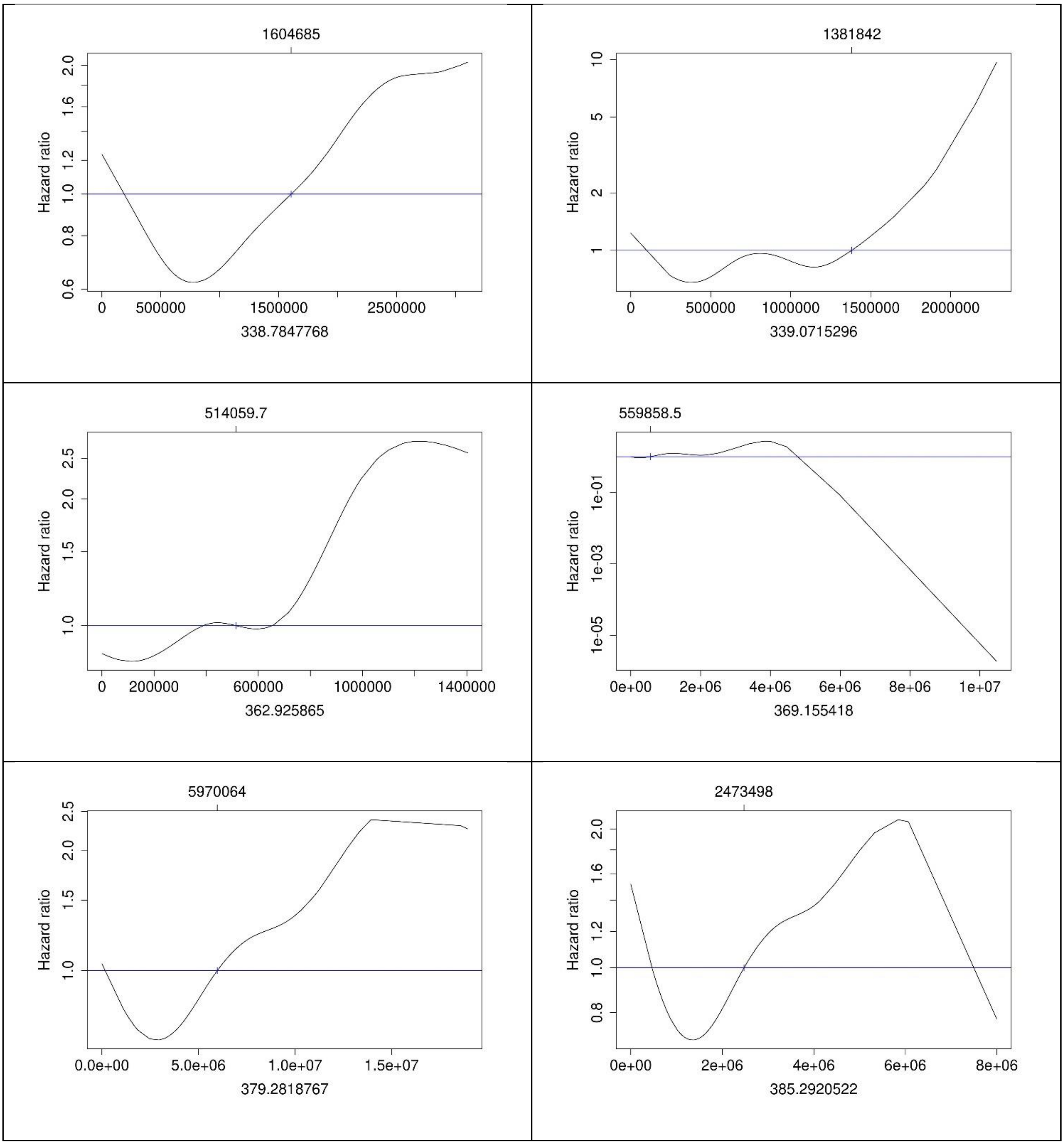

© 2025 Shen EYL et al. *JAMA Otolaryngology–Head and Neck Surgery*.

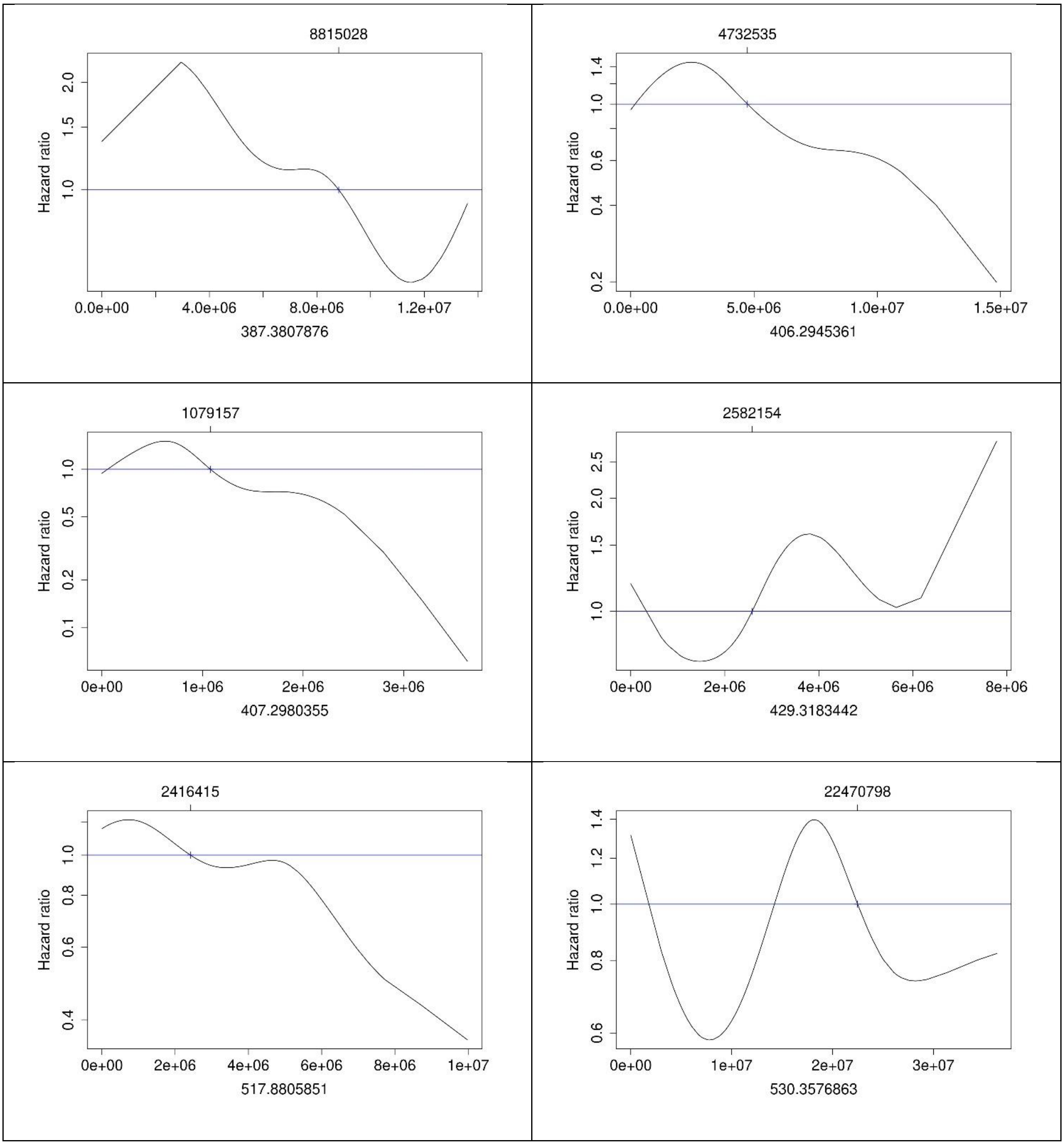

© 2025 Shen EYL et al. *JAMA Otolaryngology–Head and Neck Surgery*.

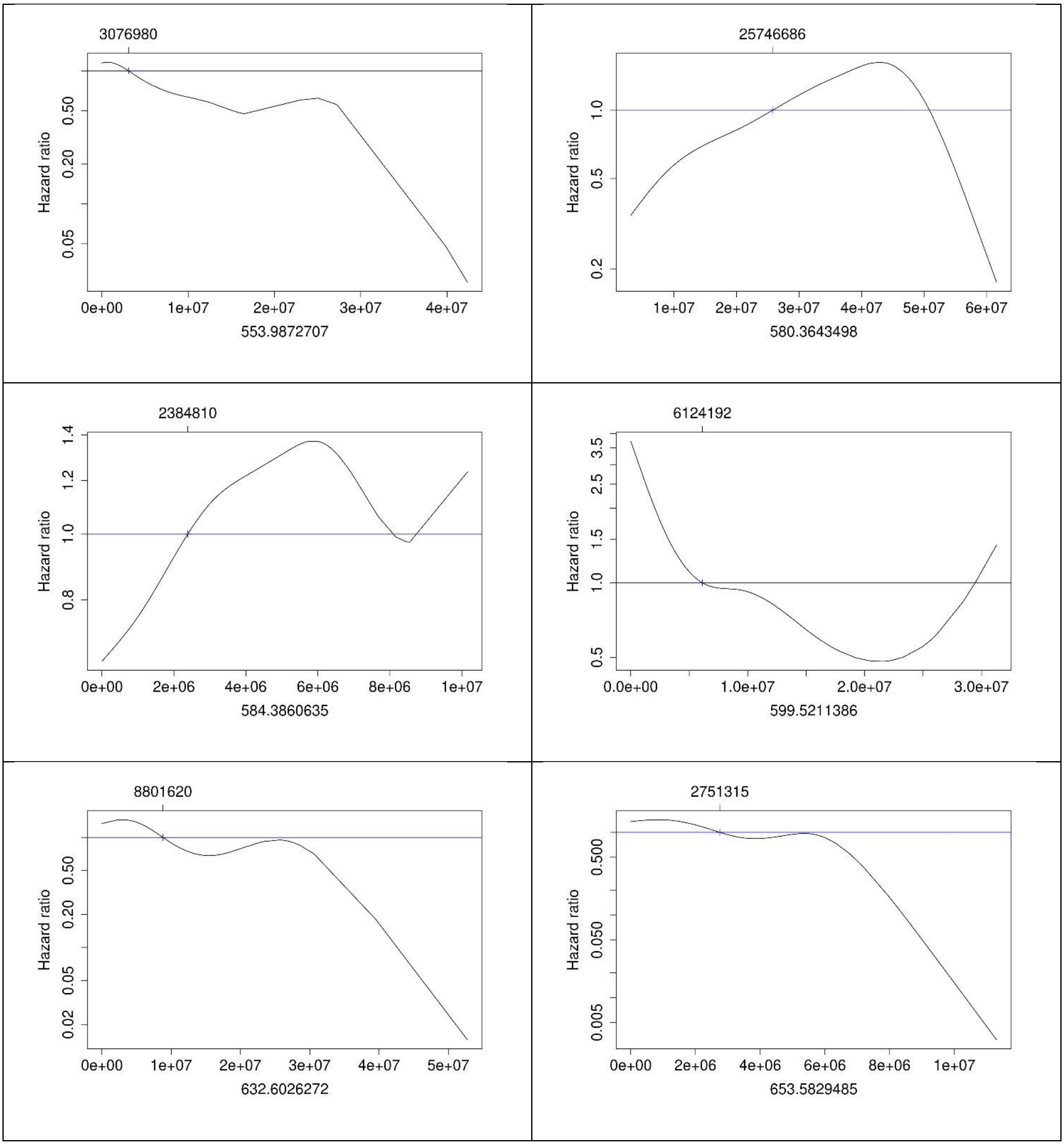

© 2025 Shen EYL et al. *JAMA Otolaryngology–Head and Neck Surgery*.

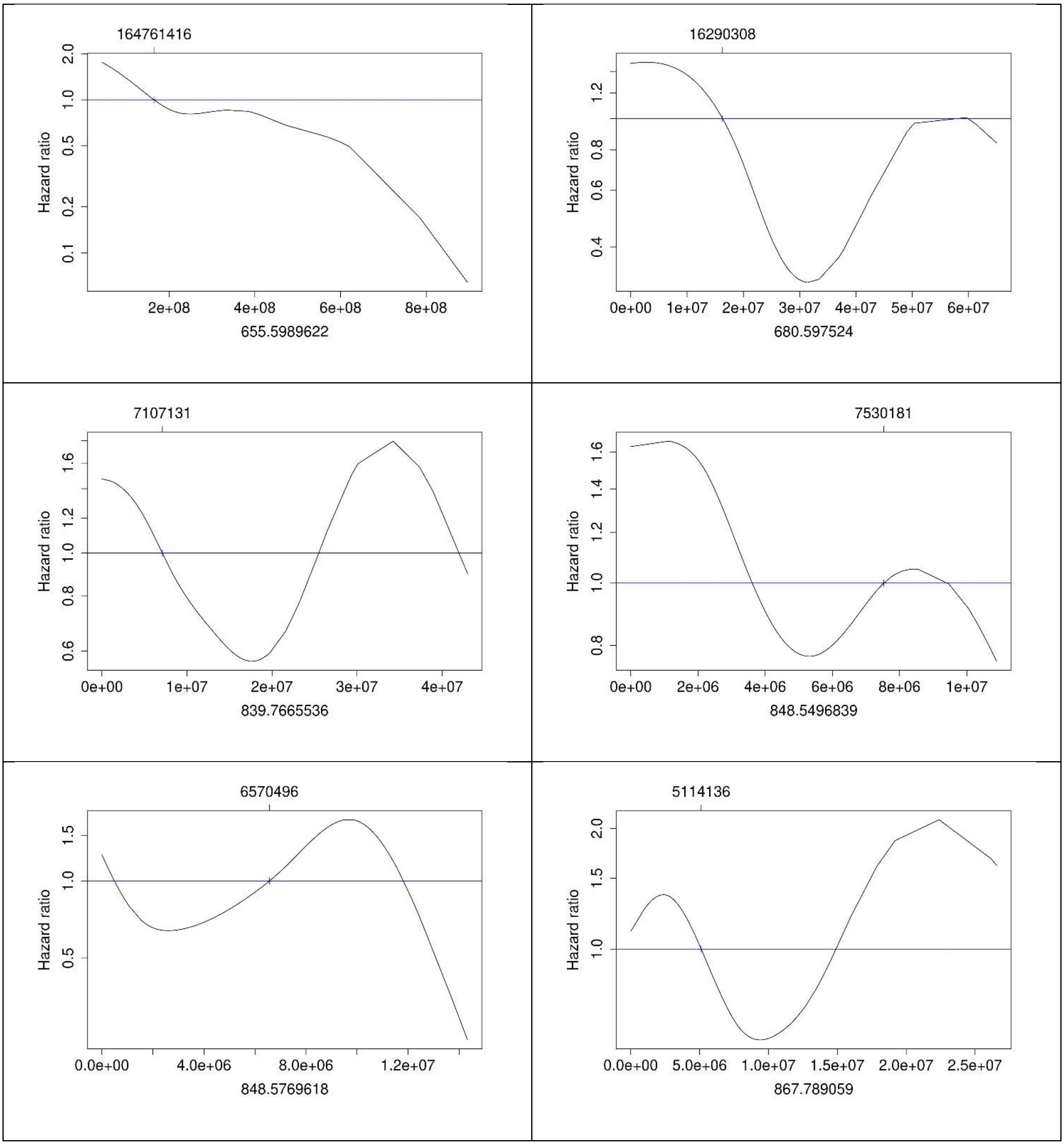

© 2025 Shen EYL et al. *JAMA Otolaryngology–Head and Neck Surgery*.

eFigure 4. DFS Kaplan-Meier Curves by Metabolite Intensity

Kaplan-Meier curves for disease-free survival based on metabolite signal intensities. The plots depict disease-free survival associated with the signal intensities of 36 metabolites, arranged by their mass-to-charge (m/z) ratio. Metabolites with signal intensities above the threshold are shown in red, whereas those below the threshold are in green. P values were calculated using the log-rank test. Each panel represents a different metabolite, with m/z values reported for each curve.

© 2025 Shen EYL et al. *JAMA Otolaryngology–Head and Neck Surgery*.

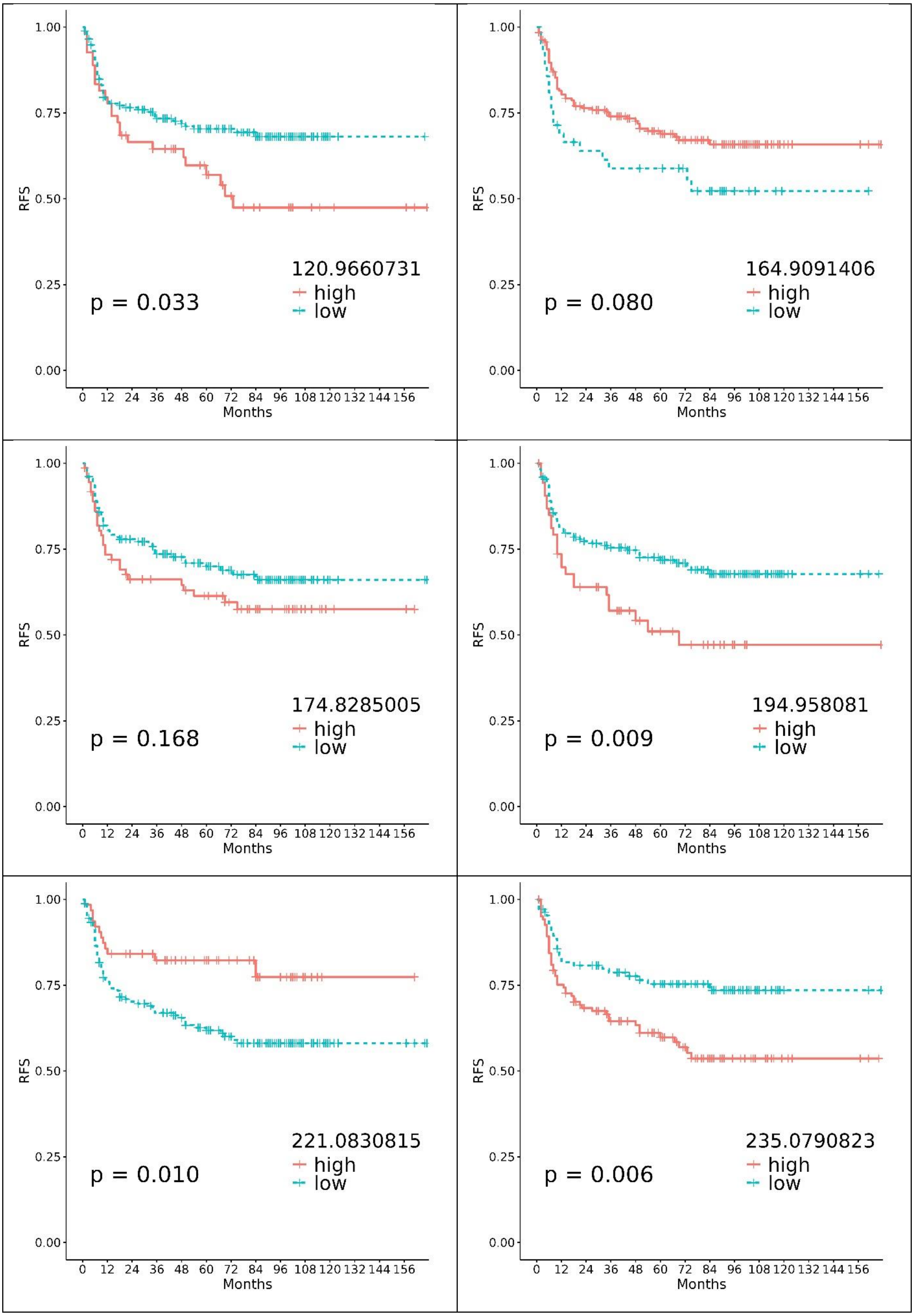

© 2025 Shen EYL et al. *JAMA Otolaryngology–Head and Neck Surgery*.

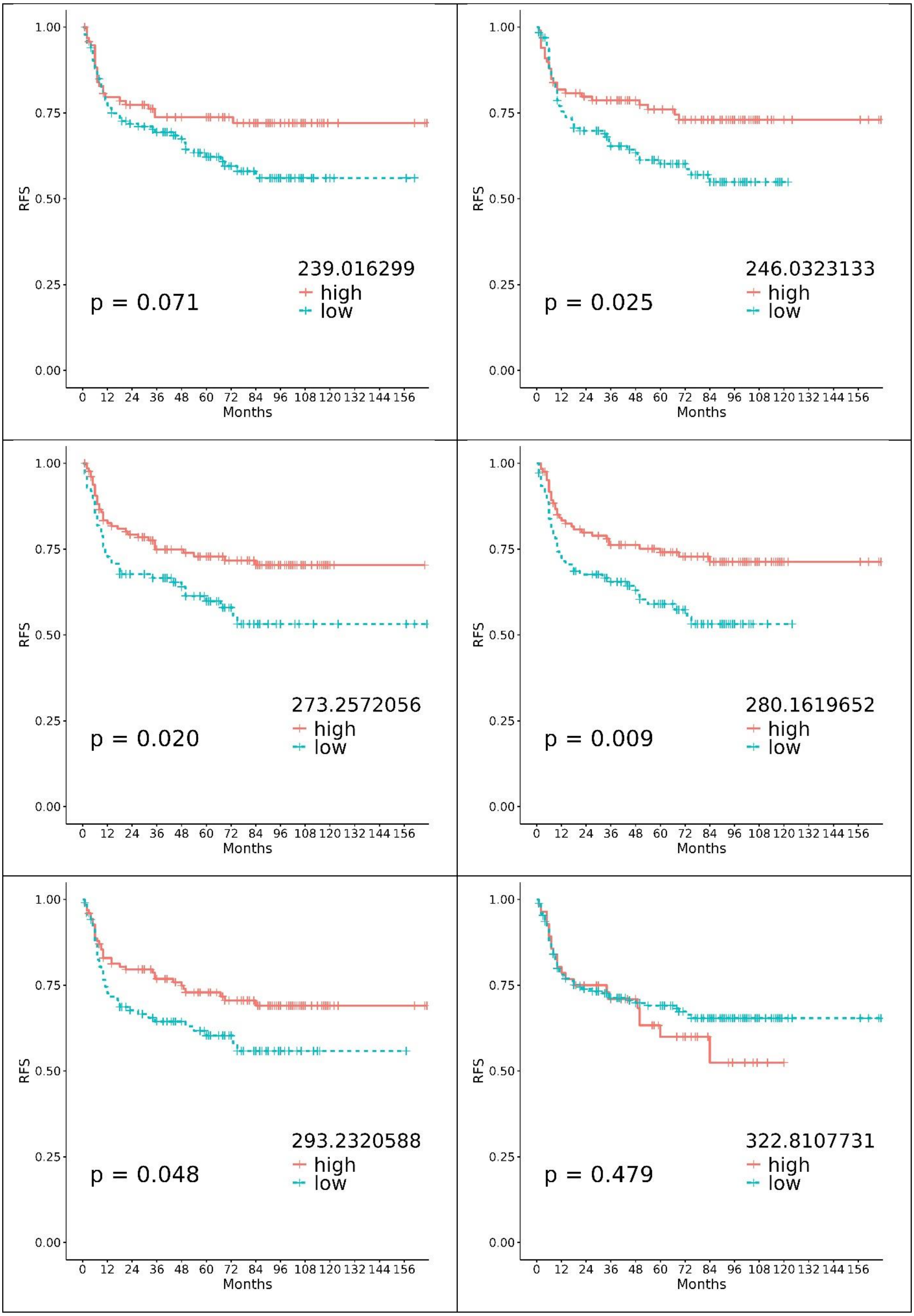

© 2025 Shen EYL et al. *JAMA Otolaryngology–Head and Neck Surgery*.

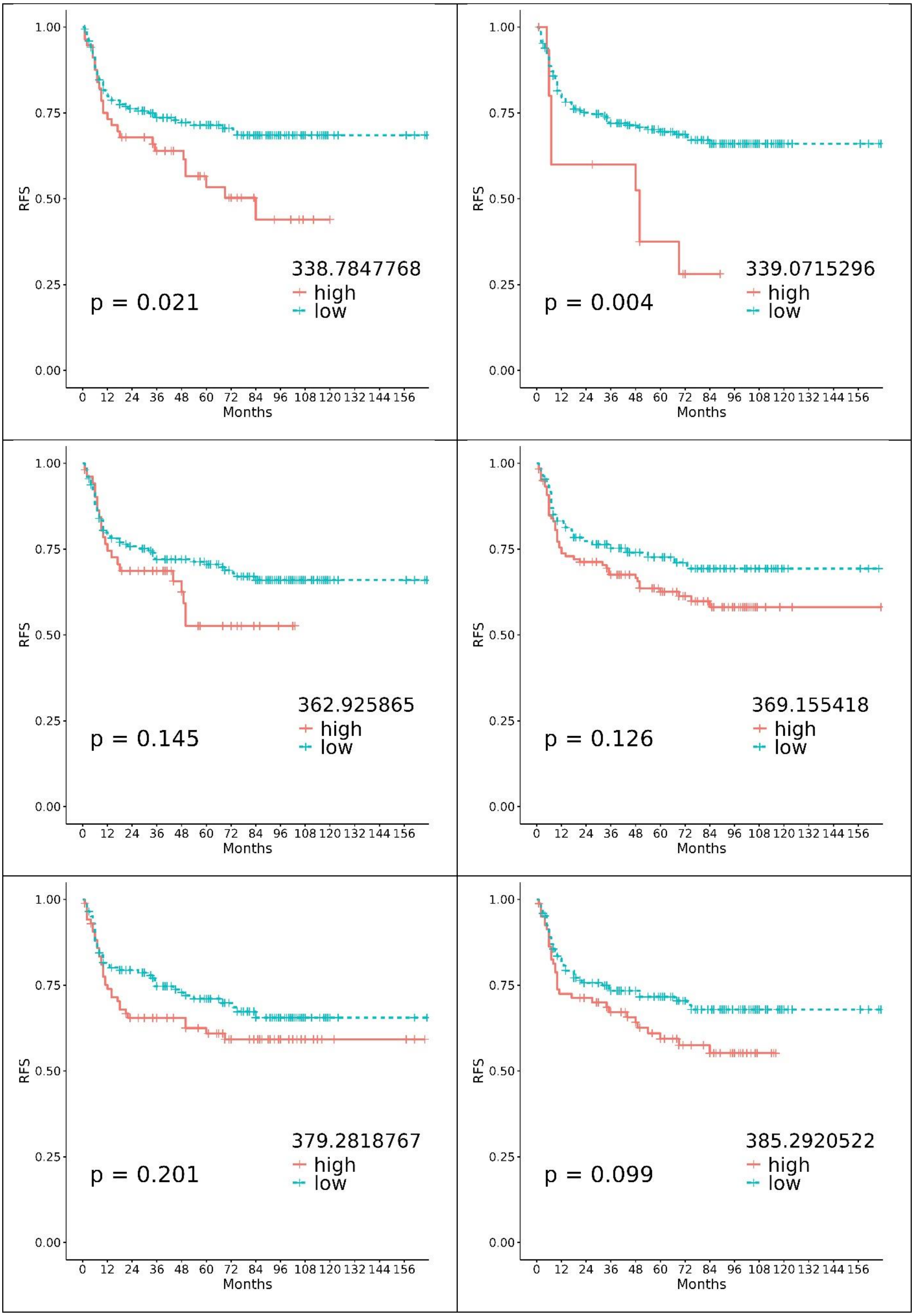

© 2025 Shen EYL et al. *JAMA Otolaryngology–Head and Neck Surgery*.

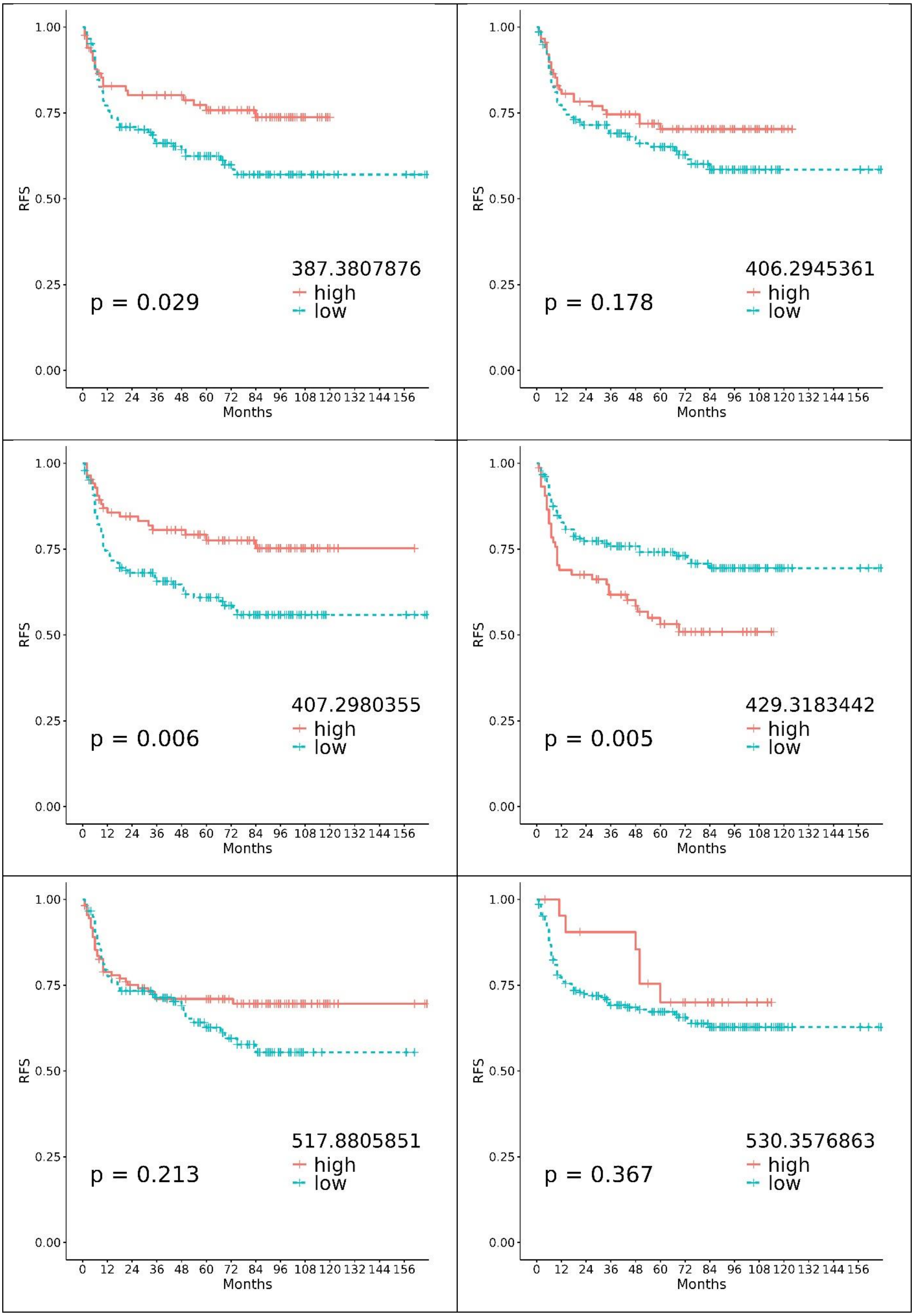

© 2025 Shen EYL et al. *JAMA Otolaryngology–Head and Neck Surgery*.

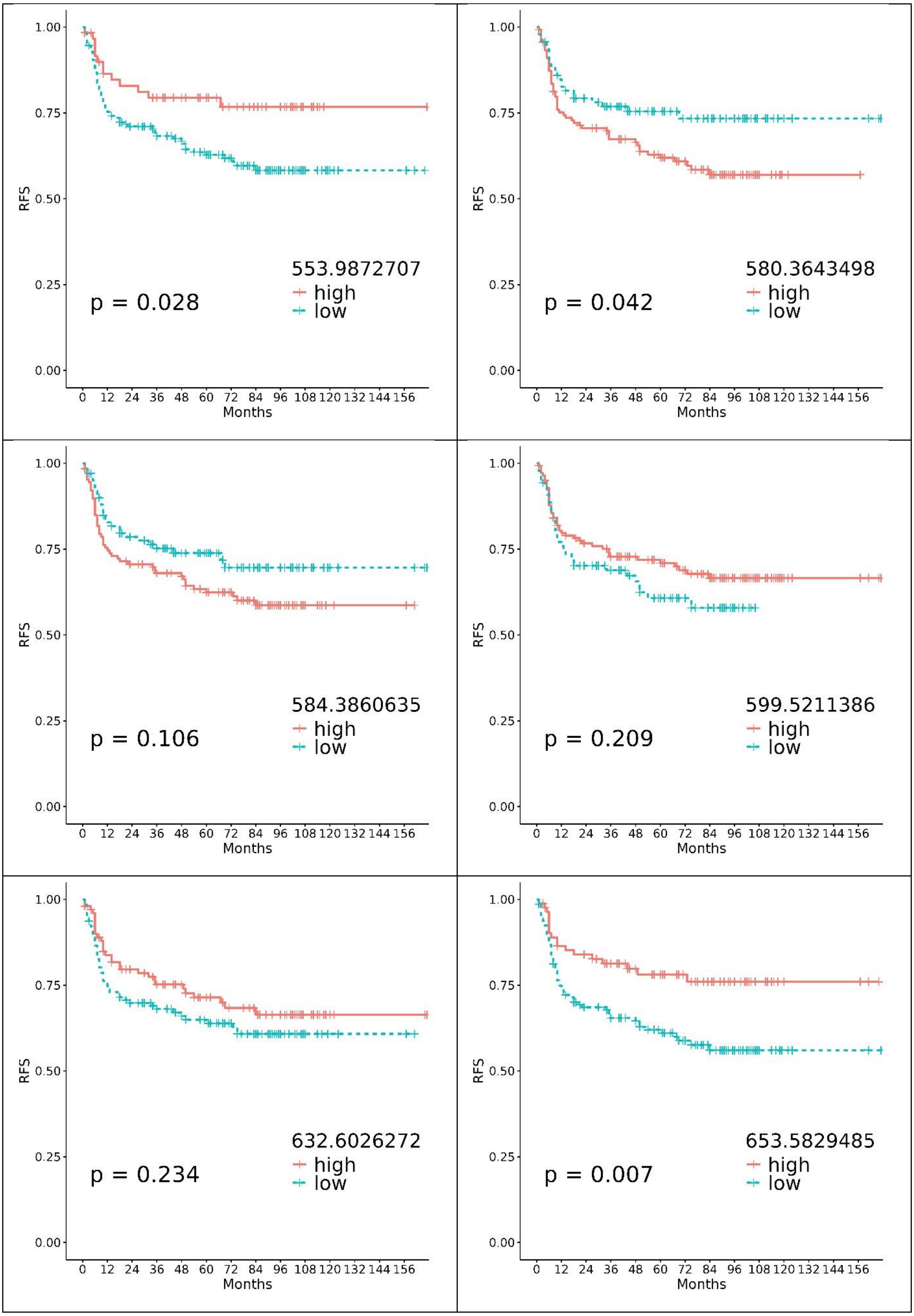

© 2025 Shen EYL et al. *JAMA Otolaryngology–Head and Neck Surgery*.

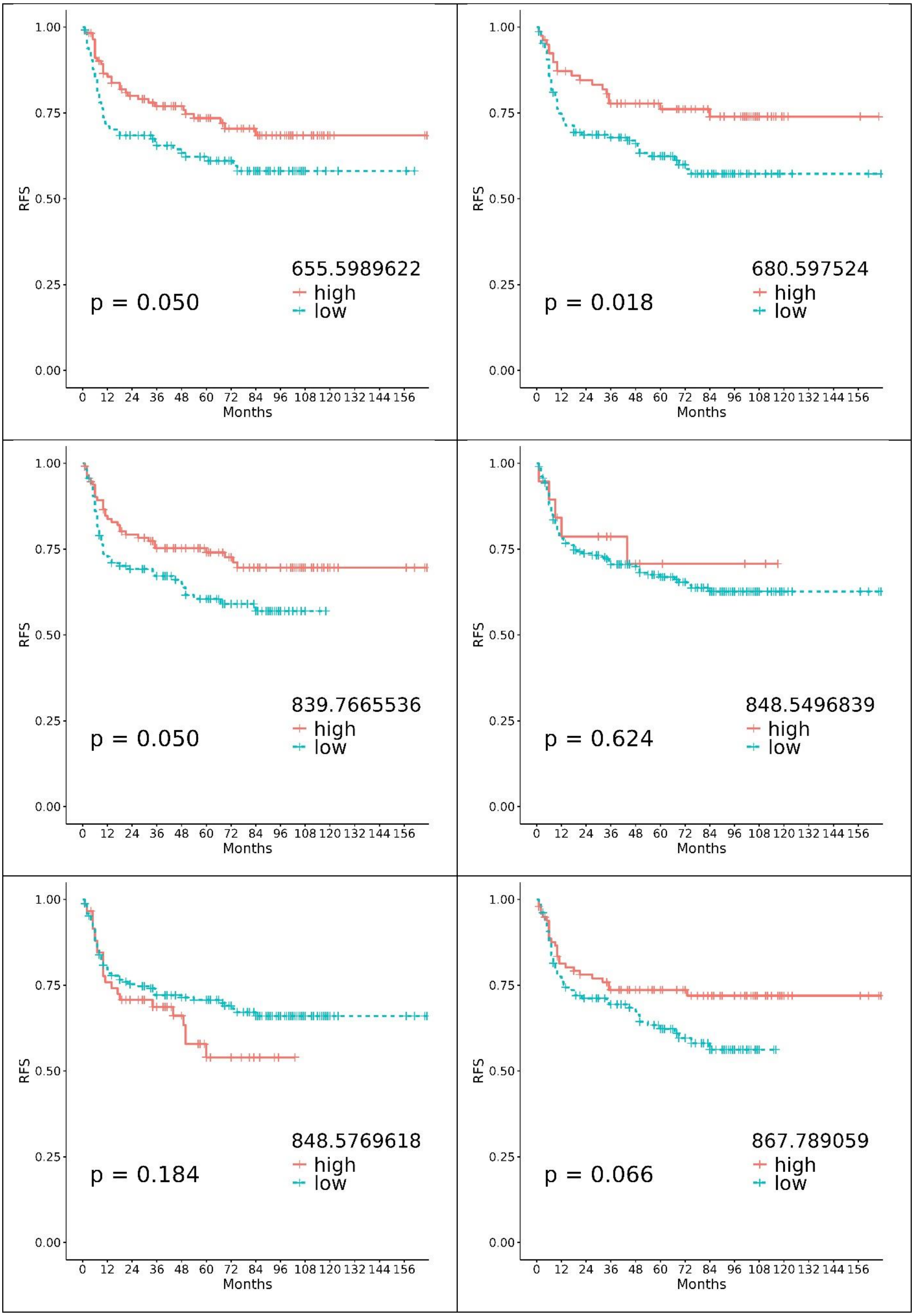

© 2025 Shen EYL et al. *JAMA Otolaryngology–Head and Neck Surgery*.

eFigure 5. AJCC Stage Specific DFS and DSS by MetaboScore

Kaplan-Meier survival curves stratified by the MetaboScore in resected OCSCC patients. (A–B) Subgroup analysis of disease-free survival (A) and disease-specific survival (B) in AJCC p-Stage III. (C–D) Subgroup analysis of disease-free survival (C) and disease-specific survival (D) in AJCC p-Stage IV.

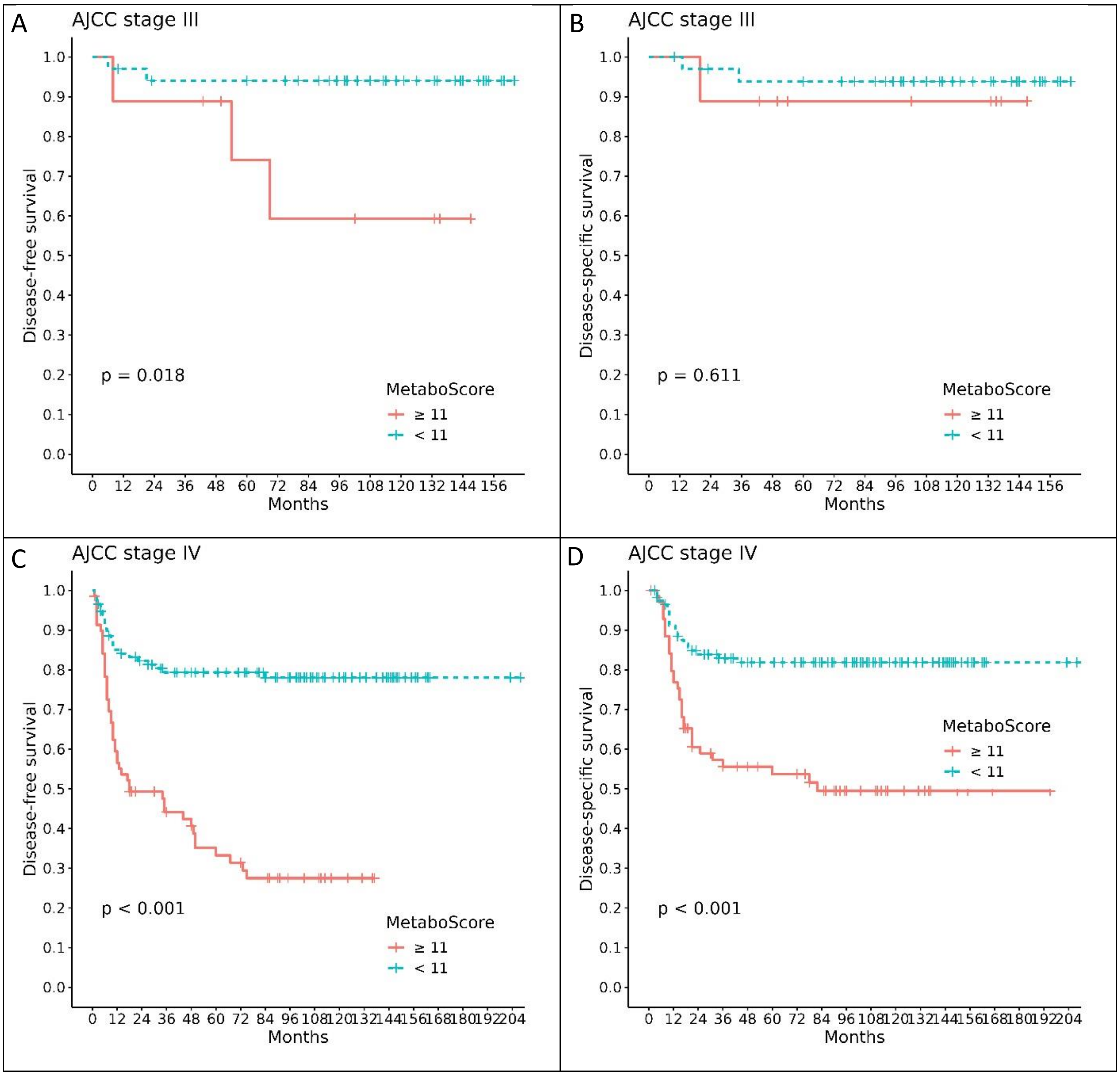

© 2025 Shen EYL et al. *JAMA Otolaryngology–Head and Neck Surgery*.

eFigure 6. pN3b Specific DFS and DSS by MetaboScore

Kaplan-Meier survival curves stratified by the MetaboScore in resected OCSCC patients. (A–B) Subgroup analysis of DFS (A) and DSS (B) in pN3b-positive patients. (C–D) Subgroup analysis of DFS (C) and DSS (D) in pN3b-negative patients. HR, hazard ratio.

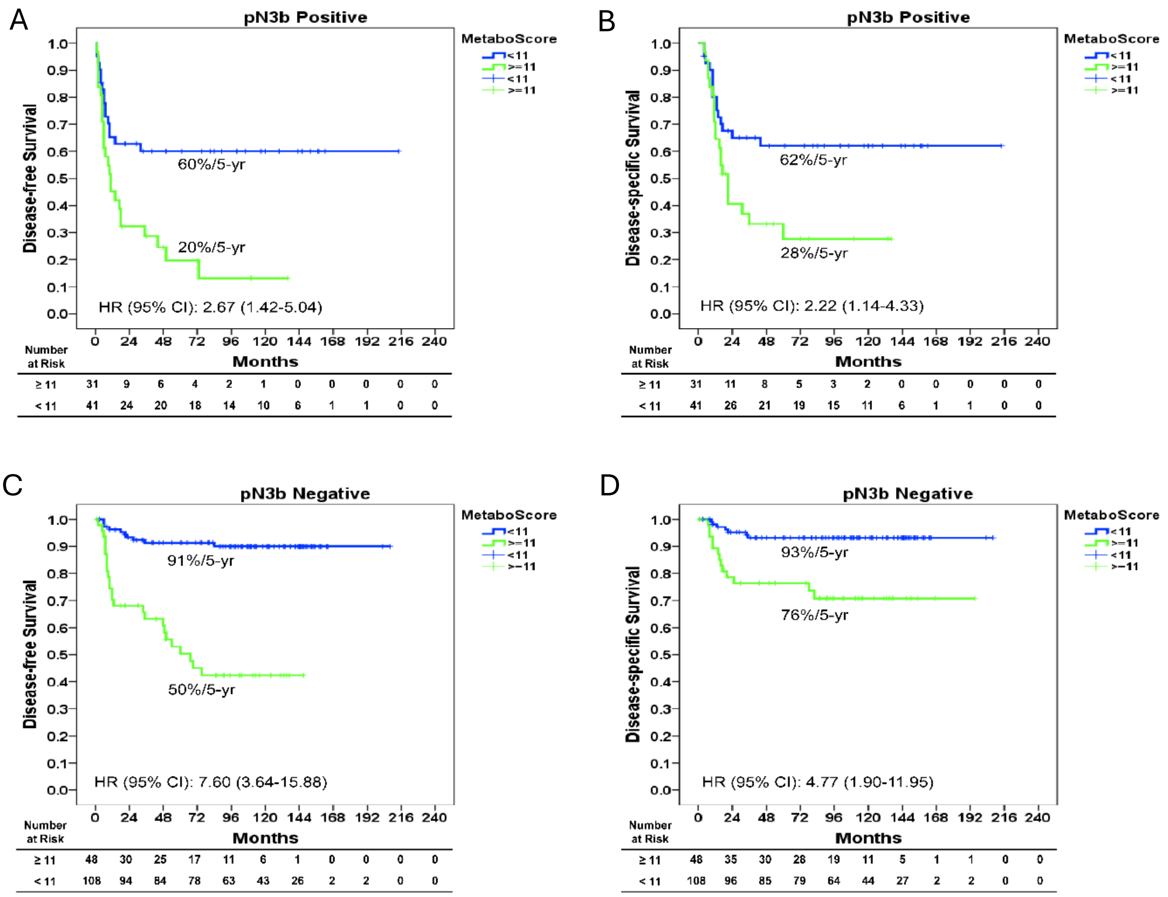

© 2025 Shen EYL et al. *JAMA Otolaryngology–Head and Neck Surgery*.

eTable. Univariable Analyses

Univariable analyses of risk factors for local control, neck control, distant metastases, disease-free survival, and disease-specific survival in patients with resected oral cavity squamous cell carcinoma (n = 228)

Risk factor	Local control<br>HR (95% CI)	Neck control<br>HR (95% CI)	Distant metastases<br>HR (95% CI)	Disease-free survival<br>HR (95% CI)	Disease-specific survival<br>HR (95% CI)
MetaboScore					
≥11	13.00 (4.90-34.00)	4.28 (1.98-9.22)	2.94 (1.67-5.16)	4.89 (3.03-7.88)	3.44 (2.01-5.88)
<11	1	1	1	1	1
Sex					
Female	1.98 (0.47-8.38)	4.37 (1.66-11.48)	2.33 (0.93-5.88)	1.83 (0.79-4.21)	2.69 (1.15-6.29)
Male	1	1	1	1	1
Age					
≥ 65 years	0.28 (0.04-2.07)	0.53 (0.13-2.24)	0.63 (0.23-1.76)	0.50 (0.20-1.23)	0.74 (0.29-1.85)
<65 years	1	1	1	1	1
Betel quid chewing					
Yes	24.7 (0.17-3559.00)	0.85 (0.29-2.43)	0.99 (0.42-2.32)	1.62 (0.71-3.74)	1.12 (0.48-2.62)
No	1	1	1	1	1
Cigarette smoking					
Yes	0.78 (0.27-2.26)	0.44 (0.19-1.04)	0.76 (0.36-1.62)	0.95 (0.49-1.85)	0.86 (0.41-1.81)
No	1	1	1	1	1
pT classification					
pT3-4	0.94 (0.28-3.12)	0.71 (0.25-2.03)	1.37 (0.49-3.79)	1.39 (0.60-3.19)	1.50 (0.54-4.13)
pT1-2	1	1	1	1	1
pN classification					
pN3	1.69 (0.76-3.78)	2.93 (1.41-6.09)	6.01 (3.31-10.90)	3.67 (2.33-5.77)	5.15 (2.97-8.92)
pN0-2	1	1	1	1	1
Pathogical stage					
pStage IV	1.83 (0.63-5.30)	7.61 (1.04-55.99)	6.49 (1.58-26.71)	4.19 (1.69-10.39)	4.88 (1.52-15.61)
pStage III	1	1	1	1	1

© 2025 Shen EYL et al. *JAMA Otolaryngology–Head and Neck Surgery*.

Tumor differentiation					
Poor	0.24 (0.03-1.80)	1.23 (0.47-3.23)	2.49 (1.34-4.62)	1.67 (0.96-2.90)	2.26 (1.25-4.08)
Well-moderate	1	1	1	1	1
Depth of invasion					
≥10 mm	1.16 (0.40-3.35)	1.12 (0.39-3.23)	1.59 (0.63-4.00)	1.61 (0.77-3.34)	1.86 (0.74-4.65)
<10 mm	1	1	1	1	1
Margin status					
≤4 mm	1.12 (0.48-2.53)	1.36 (0.63-2.93)	1.59 (0.90-2.82)	1.26 (0.78-2.04)	1.36 (0.78-2.36)
>4 mm	1	1	1	1	1
Bone marrow invasion					
Yes	1.13 (0.39-3.28)	0.62 (0.19-2.06)	1.61 (0.82-3.14)	1.30 (0.73-2.32)	1.56 (0.82-2.95)
No	1	1	1	1	1
Skin invasion					
Yes	0.67 (0.16-2.82)	0.59 (0.14-2.46)	1.09 (0.47-2.56)	0.80 (0.37-1.74)	0.97 (0.42-2.26)
No	1	1	1	1	1
Perineural invasion					
Yes	1.01 (0.47-2.15)	1.53 (0.72-3.24)	1.52 (0.86-2.69)	1.38 (0.87-2.18)	1.71 (0.99-2.95)
No	1	1	1	1	1
Lymphatic invasion					
Yes	3.92 (0.92-16.82)	5.99 (2.25-15.94)	7.09 (3.38-14.85)	5.47 (2.77-10.77)	7.61 (3.78-15.34)
No	1	1	1	1	1
Vascular invasion					
Yes	2.22 (0.52-9.52)	2.82 (0.97-8.14)	3.32 (1.55-7.09)	3.06 (1.56-5.98)	4.10 (2.06-8.16)
No	1	1	1	1	1
Treatment modality					
Surgery+RT/CRT	1.09 (0.38-3.14)	1.41 (0.43-4.66)	8.35 (1.15-60.50)	2.46 (0.99-6.10)	9.48 (1.31-68.52)
Surgery alone	1	1	1	1	1

Abbreviation: HR, hazard ratio; CI, confidence interval; RT, radiotherapy; CRT, chemoradiotherapy

© 2025 Shen EYL et al. *JAMA Otolaryngology–Head and Neck Surgery*.
