# Supplementary material for: Serum Metabolomics for Prognostic Stratification in Resected Advanced-Stage Oral Cavity Cancer
Source: JAMA Otolaryngol Head Neck Surg. 2025 Dec 4;152(2):172–81. doi: 10.1001/jamaoto.2025.4267 (PMC12679420; doi:10.1001/jamaoto.2025.4267)
Supplement: Supplement 2. — Data Sharing Statement [file jamaotolaryngolheadnecksurg-e254267-s002.pdf]

Data Sharing Statement

Shen. Serum Metabolomics for Prognostic Stratification in Resected Advanced-Stage Oral Cavity Cancer. *JAMA Otolaryngol Head Neck Surg*. Published December 04, 2025. doi:10.1001/jamaoto.2025.4267

Data
Data available: No
